# Functional Glyco-Nanogels for Multivalent Interaction with Lectins

**DOI:** 10.3390/molecules24101865

**Published:** 2019-05-15

**Authors:** Jo Sing Julia Tang, Sophia Rosencrantz, Lucas Tepper, Sany Chea, Stefanie Klöpzig, Anne Krüger-Genge, Joachim Storsberg, Ruben R. Rosencrantz

**Affiliations:** 1 Fraunhofer Institute for Applied Polymer Research IAP, Biofunctionalized Materials and (Glyco)Biotechnology, Geiselbergstr. 69, 14476 Potsdam, Germany; josing.tang@iap.fraunhofer.de (J.S.J.T.); sophia.rosencrantz@iap.fraunhofer.de (S.R.); lucas.tepper@iap.fraunhofer.de (L.T.); sany.chea@iap.fraunhofer.de (S.C.); 2 Fraunhofer Institute for Applied Polymer Research IAP, Biomaterials and Healthcare, Geiselbergstr. 69, 14476 Potsdam, Germany; stefanie.kloepzig@iap.fraunhofer.de (S.K.); anne.krueger-genge@iap.fraunhofer.de (A.K.-G.); joachim.storsberg@iap.fraunhofer.de (J.S.)

**Keywords:** nanogels, glycans, lectins, multivalency, glycogels, glycopolymers, precipitation polymerization

## Abstract

Interactions between glycans and proteins have tremendous impact in biomolecular interactions. They are important for cell–cell interactions, proliferation and much more. Here, we emphasize the glycan-mediated interactions between pathogens and host cells. *Pseudomonas aeruginosa*, responsible for a huge number of nosocomial infections, is especially the focus when it comes to glycan-derivatives as pathoblockers. We present a microwave assisted protecting group free synthesis of glycomonomers based on lactose, melibiose and fucose. The monomers were polymerized in a precipitation polymerization in the presence of NiPAm to form crosslinked glyco-nanogels. The influence of reaction parameters like crosslinker type or stabilizer amount was investigated. The gels were characterized in lectin binding studies using model lectins and showed size and composition-dependent inhibition of lectin binding. Due to multivalent presentation of glycans in the gel, the inhibition was clearly stronger than with unmodified saccharides, which was compared after determination of the glycan loading. First studies with *Pseudomonas aeruginosa* revealed a surprising influence on the secretion of virulence factors. Functional glycogels may be in the future potent alternatives or adjuvants for antibiotic treatment of infections based on glycan interactions between host and pathogen.

## 1. Introduction

A vast number of pathogens utilize glycan-mediated interactions for cell invasion and their actual pathogenicity [1,2]. Well known examples are enterohemorrhagic *E. coli* (EHEC) with its shiga-like toxin [3], *Clostridium difficile* with its glycan binding Toxin A [4,5], *Vibrio cholera* [6] and others. Several small molecules based on sugars as well as larger glycoclusters have been synthesized as patho-blocking agents for fighting these microbes or their toxins [7,8,9].

A key point for strong glycan-mediated interactions is the multivalent presentation of glycan ligands inducing the ‘cluster glycoside effect’ [10,11,12]. Good choices for multivalent glycostructures are so called glycopolymers [13,14,15,16]. These are polymers with pendent glycan groups attached to a polymeric backbone. Glycopolymers have been shown to enable very high binding avidities with lectins resulting in K_D_ values in the nanomolar range [17]. Multivalency is crucial for good interactions between glycans and lectins and may increase binding strength in orders of magnitude. Examples of the usage of glycopolymers are biosensor surfaces for lectin binding studies or as mannose-based scavenging material for *E. coli* [17,18,19,20,21].

Recently, polymeric gels containing glycans were synthesized [22] via a microfluidic set-up to yield lactose containing gels with good binding to appropriate lectins [23]. Micro-, nano- or hydrogels in general can be considered as very promising systems for lectin binding. This is mainly due to their swollen “waterlike” state, their biocompatibility, the large internal volume and their potential multivalent presentation mode with incorporated glycans [24,25]. A very often used monomer for nanogel synthesis is NiPAm (*N*-isopropylacrylamide), which yields thermoresponsive polymers and enables precipitation polymerization of uniform gel particles [26,27]. PNiPAm (Poly(*N*-isopropylacrylamide)) shows a lower critical solution temperature (LCST) of about 32 °C. Below this temperature, gel particles are considered swollen and rather fuzzy, whereas above the LCST the particles become more defined, smaller and more rigid due to denser packaging. While NiPAm itself is considered cytotoxic, PNiPAm is reported to be biocompatible and non-toxic to cells [28,29,30]. The thermoresponsive properties of PNiPAm enable the batch synthesis of gels via precipitation polymerization preventing the usage of organic solvents compared to, e.g., emulsion polymerization.

*Pseudomonas aeruginosa* (PA) is an opportunistic pathogen rated as critical by the WHO list indicating for which strain new antibiotics are urgently needed [31,32]. Interestingly, PA utilizes two lectins (LecA and LecB) as virulence factors [33,34]. Many glycan derivatives were synthesized as patho-blocking agents [35,36]. However, the number of reports on glycopolymeric multivalent structures for lectin inhibition is rather limited. Potent glycomaterials must comprise a sufficient multivalent mode of ligand presentation. For PA, it was shown that multivalent ligands based on glycooligomers, dendrimers or as peptide derivatives are superior to the monovalent species [37,38,39,40,41]. This stands also for other lectins: Here, increase of affinity over several orders of magnitude by multivalent ligand presentation is known [13].

We here describe for the first time the synthesis of different glycogel species containing either lactose (Galβ1,4Glc-), melibiose (Galα1,6Glc-) or fucose. The glycans were chosen as readily available, naturally occurring structures, with the latter two known to act as ligand for LecA and LecB [42,43]. By enabling multivalent presentation in the gel, we expect to circumvent the necessity of introducing modifications to monovalent glycans increasing their affinity. The gels were synthesized in a batch process via precipitation polymerization utilizing NiPAm and lactose, melibiose or fucose glycomonomers in the presence of crosslinker and surfactant for stabilization. In this study, we focus on the influence of synthesis parameters on the inhibition potential of the gels and determined the presence or absence of a multivalent effect compared to monovalent, soluble sugars. Ability of lectin inhibition was screened by an ELISA-type approach with fluorescently labeled plant lectins as model lectins. Ultimately, we tested in a preliminary study the influence of the gels on the growth of PA.

In the future, glycan-based soft matter can be a good way to yield biocompatible yet strong pathoblockers for medical applications. Glycoscavengers can be used for numerous different pathogens and be a promising alternative to antibiotic treatment with minimal selection pressure avoiding acquirement of resistances.

## 2. Results and Discussion

### 2.1. Synthesis of Glycomonomers

**Scheme 1:**
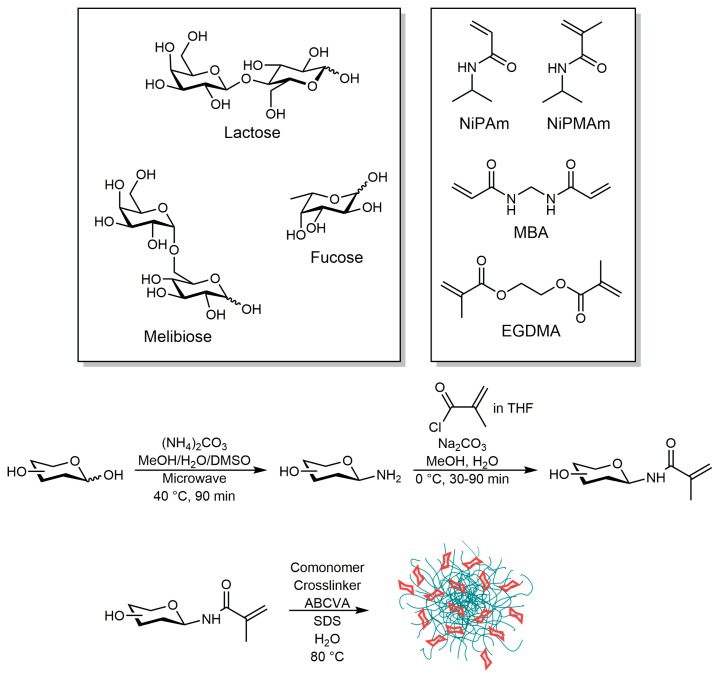
Lactose, melibiose and fucose were converted via a protecting group free synthesis utilizing microwave irradiation to the respective methacrylamides. The monomers were used for the synthesis of glyco-nanogels in a precipitation polymerization in the presence of comonomer *N*-isopropylacrylamide (NiPAm) or *N*-isopropylmethacrylamide NiPMAm and crosslinker methylenebis(acrylamide) (MBA) or ethylene glycol dimethacrylate (EGDMA).

For the synthesis of glycomonomers with a polymerizable moiety at the C1-position we chose a protecting group free microwave-assisted Kochetkov-amination with subsequent reaction with methacryloylchloride (Scheme 1) [44,45,46,47].

Modification of the saccharides at C1-postion should not affect the biological recognition of the sugar by lectins. The disaccharides lactose and melibiose as well as the monosaccharide fucose were used as starting material and converted to the respective methacrylamides MelMAm (melibiose-methacrylamide), LacMAm (lactose-methacrylamide) and FucMAm (fucose-methacrylamide) (Scheme 1). The overall yields ranged from 18% to 75%, which is sufficient for the production of nanogels. The compounds were identified by NMR spectroscopy and ESI MS (Figures S1–S9, Supporting Information). Advantages of the synthesis are the usage of cheap starting materials, the intact cyclisation of the reducing sugar and the regioselectivity for the C1-position. However, it must be noted that the β-anomer is strongly favored as reaction product. For melibiose and lactose, we do not expect any drawbacks regarding this, but β-fucose is a rather rare compound and may not be recognized by typical fucose binding lectins like *Ulex europaeus* agglutinin I (UEA I). However, it is reported that LecB binding can be inhibited to some extent by fucosylamine, which is in fact 1-amino-β-l-fucose and the intermediate of our synthesis route [35,43,48].

### 2.2. Synthesis of Glycogels

#### 2.2.1. Free-Radical Precipitation Polymerization

We evaluated two different procedures for the preparation of the glycogels: Inverse emulsion polymerization and free-radical precipitation polymerization with NiPAm and NiPMAm. The yields of the emulsion polymerization turned out to be not sufficient for subsequent analysis in lectin-assays or tests with PA (yields were below 5%, data not shown). We assume a slow propagation and deactivation by the glycomonomers resulting in these low yields. The syntheses via free-radical precipitation polymerization in contrast gave a sufficient yield of up to 75% compared to the initially used amount of monomer and crosslinker. Furthermore, conducting the reaction in water gives the advantage of bypassing the poor solubility of the unprotected glycomonomers in organic solvents and enabling a “green” route avoiding potentially toxic solvents. Hence, we synthesized all gels presented here by free-radical precipitation polymerization in water. The reaction temperature was chosen to be 80 °C as this is above the LCST of PNiPAm/PNiPMAm as well as above the 10 h half-life decomposition temperature of the water soluble azoinitiator 4,4′-azobis(4-cyanovaleric acid) (ABCVA). The freeze-dried glycogels were hygroscopic and TGA analysis revealed an equilibrium water content of about 10%. Throughout the text the gels carrying melibiose are labeled “MG”, gels with lactose are labeled “LG” and fucose containing gels are labelled “FG”. Gels without sugar serve as comparative sample and negative control for the bioassays and are labelled with “G”.

#### 2.2.2. Comonomer and Crosslinker

Since the glycopolymer itself does not precipitate in water upon chain propagation, we required comonomers which are water soluble and exhibit a LCST as a polymer. Furthermore, the different monomers should have similar reaction kinetics in order to form a hydrogel with evenly distributed glycosides to the greatest extent possible. As the glycomonomers are methacrylamides, we selected the methacrylamide NiPMAm and ethylene glycol dimethacrylate (EGDMA) and compared the performance to the acrylamide NiPAm and *N*,*N*′-methylenebis(acrylamide) (MBA) as comonomer and crosslinker, respectively. Interestingly, PNiPMAm glycogels (MG-7) proved to be unsuitable for binding assays, as the pure PNiPMAm nanogel (G-3) itself seems to influence the lectin binding (see Section 2.3). Hence, no reliable lectin binding data can be produced with PNiPMAm gels and we omitted these gels. Gels containing PNiPAm showed clearly better suitability for the lectin assays. The type of crosslinker (MBA vs. EGDMA) had no significant influence on the yield but for glycogels synthesized with NiPAm and EGDMA (MG-8), binding studies with lectins showed less inhibition potentials (see Section 2.3) than glycogels produced with NiPAm and MBA. Thus, we chose for the syntheses of glycogels NiPAm as the comonomer enabling the precipitation polymerization, and MBA as crosslinker. We assume that for good inhibition performance, a core-shell-like gel morphology is appreciated where the core is build up by the non-glycosylated monomers, surrounded by a glycan-shell. This can be achieved most likely by using the fast polymerizing acrylamides NiPAm and MBA together with the rather slow methacrylamide glycomonomers.

#### 2.2.3. PNiPAm Glycogels

**Figure 1:**
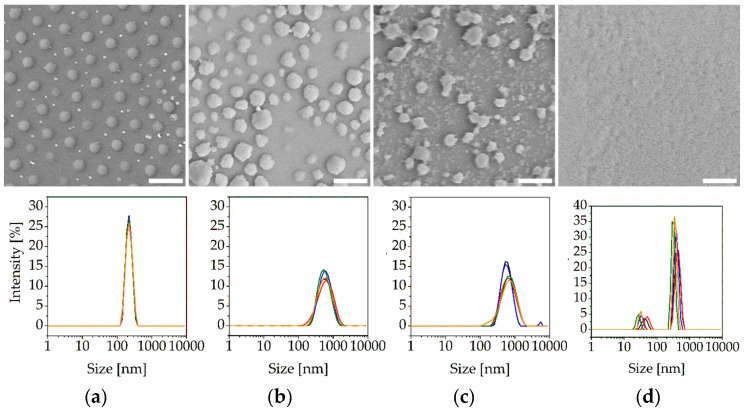
Scanning electron microscopy (SEM) images and dynamic light scattering (DLS) size distribution by intensity (at 50 °C) of different nanogels: (**a**) monodisperse PNiPAm nanogel particles G-1 synthesized with 0.2 mM SDS; (**b**) glycogel MG-1 synthesized with 0.4 mM SDS; (**c**) MG-2 synthesized with 2.0 mM SDS; (**d**) glycogel MG-3 synthesized with 4.0 mM SDS. Scale bars represent 1 µm.

Typically, when PNiPAm nanogels particles are formed by precipitation polymerization, the reaction solution turns turbid. The turbidity depends on the concentration and size of the particles. For the glycogels, we observed a strikingly lower turbidity during reaction. This indicates that the gels were not only consisting of NiPAm but the glycomonomer could be incorporated into the polymers as well. We assume that the glyco-comonomer interferes with the complete collapse of PNiPAm and forms a fuzzy shell-like structure around a PNiPAm core. In scanning electron microscopy (SEM) as well as in atomic force microscopy (AFM) images, we can find spherical particles (see Supporting Information). Hence, we achieved glycogel particles and not free polymers. From dynamic light scattering (DLS) measurements and SEM images, we observe high polydispersity in contrast to the excellent monodispersity of pure PNiPAm nanogels (Figure 1). Due to the hydrophobic propyl moiety of NiPAm, the polymer precipitates above its LCST when the hydrophobic interactions dominate. The glycomonomers exhibit a high hydrophilicity. This property may counteract the hydrophobic interactions of NiPAm which could explain the high polydispersity. As the gels do not dissolve in water independently of the surrounding temperature, we can assume that the products are crosslinked networks and not free copolymer chains.

We used SDS in order to stabilize the gels during reaction. At similar surfactant concentrations, the sizes of the glycogels are larger than the size of the PNiPAm nanogels (Figure 1), which may be related to the fuzzy glyco-shell, which tends to swell in aqueous media independent of the surrounding temperature. Furthermore, with increasing surfactant concentration, the size of the glycogels do not evidently become smaller and their monodispersity does not increase either (Table 1). Typically, increased concentrations of stabilizer in precipitation polymerizations lead to smaller diameters [49]. The effect of SDS seems to be diminished in the case of glycogels. Surprisingly, high concentrations of SDS appear to even increase the polydispersity. We assume that the hydrophilic property of the glycomonomers counteracts the formation of monodisperse and uniform particles. A possible explanation is that the hydrophilic part of the surfactant and the hydrophilic glycomonomer repell each other, which disturbs the formation of stabilizing SDS-corona around the growing gel particles. Therefore, a high amount of SDS may cause the glycosyl moiety to distribute and scatter in the water instead of letting formed polymer chains immediately collapse into a coil in between the surfactants.

Temperature-dependent DLS measurements show that some glycogels still retain some of the thermoresponsiveness of PNiPAm. It has to be noted that the hydrodynamic diameter (D_h_) of the glycogels are not reliable as the polydispersity index (PDI) is quite high. However, we can observe a trend where the PDI decreases at 50 °C as well as the averaged hydrodynamic diameter (Table 2).

**Figure 2:**
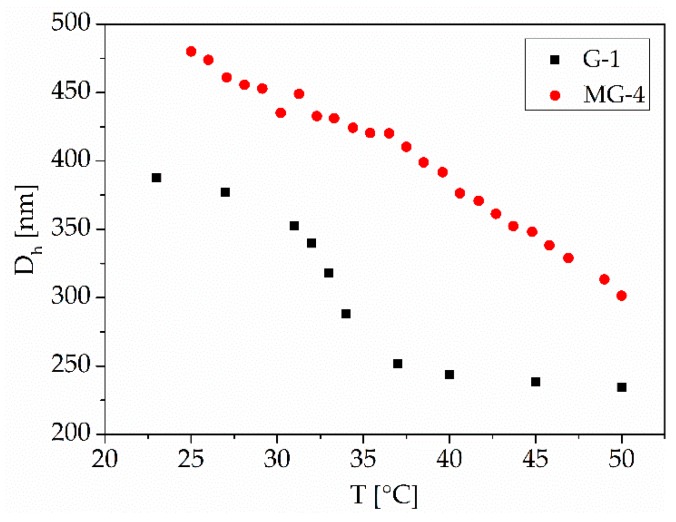
Temperature-dependent analysis of the hydrodynamic diameter of PNiPAm nanogel G-1 and melibiose glycogel MG-4 using DLS. While still thermoresponsive to some extent, no distinct lower critical solution temperature (LCST) can be detected for the glycogels (MG-4).

This comes in agreement with the typical behavior of PNiPAm nanogels. Their hydrodynamic diameter decreases with increasing temperature as they collapse. Their PDI usually improves at temperatures above the LCST since the collapsed particles with the defined surface border are easier to measure via DLS than swollen, soft nanogels with their fuzzy surface and dangling polymer chains. Though, we do not observe a defined LCST for the glycogels (see Figure 2).

#### 2.2.4. Initiation of the Polymerization

Generally, the synthesis of pure PNiPAm nanogels require only little amounts of initiator (0.25 mol%) [50]. By using a mixture of NiPAm and MelMAm, 0.25 mol% of initiator seems to be insufficient as the reaction mixture stays clear. This is even the case with the fourfold amount of ABCVA (1 mol%). This indicates that no crosslinked networks are formed or that the polymerization is not taking place or being inhibited somehow. Therefore, we fed an additional amount initiator after two hours to the reaction mixture (Table 3, MG-1). It is also possible to use an even higher amount of the initiator from the beginning to start the reaction (MG-4). For better comparison with previously synthesized gels, we continued with the method mentioned first with an initiator feed. Preceding equilibration of the reaction mixture at the reaction temperature, as often executed for PNiPAm nanogels [49], does not lead to significantly better binding performance (MG-5). The method of reaction start does not significantly affect the yield, hydrodynamic diameter nor the polydispersity. Thus, we synthesized the rest of the glycogels without temperature equilibration.

#### 2.2.5. Glycogels with Various Crosslinking Densities

The crosslinking density generally influences the morphology of PNiPAm nanogels such as deformability, softness and swelling ability [50]. Here, we compare glycogels based on different saccharides and the influence of the crosslinker amount. The highest yield of 75% related to the initial amount of total monomer was achieved with LacMAm and 5 mol% crosslinker resulting in glycogel LG-1 (Table 4). Similar synthesis with MelMAm and 5 mol% crosslinker gained 29% less yield (MG-6; 46%). For melibiose glycogels, the highest yield was achieved with 10 mol% of crosslinker (MG-2; 67%). Comparable synthesis with FucMAm and 5 mol% crosslinker also achieved lower yields of 56% (FG-1). When changing the crosslinking density from 5 to 10 mol% (FG-2), the yields dropped to 33%. It has to be noted that during synthesis the fucose glycogels precipitated more readily than the other glycogels and tend to aggregate during reaction. Here, we filtered off the large aggregated sediments before freeze-drying. We assume that higher amounts of crosslinker lead to more aggregation. This is consistent with the low yield of FG-2. It is strongly evident how differently various crosslinker amounts and various saccharides influence the precipitation polymerization, even though LacMAm and MelMAm are both disaccharides. Melibiose exhibits a higher water solubility than lactose, indicating a stronger negative influence on the precipitation behavior of the gels. Therefore, in case of melibiose glycogels, higher amounts of crosslinker might be necessary in order to gain higher yields since MBA reacts faster than NiPAm and, thus, does not slow down the reaction. Fucose, however, carries a non-polar methyl-group which can reestablish a more PNiPAm-like precipitation behavior, hence, the reaction solution turned turbid faster. Besides, the higher hydrophobicity of fucose might be a cause for the aggregation during synthesis. 

#### 2.2.6. Amount of Incorporated Carbohydrates in Glycogels

We determined the amount of incorporated glycomonomer by a phenol-sulfuric acid assay and studied how it is influenced by synthesis parameters and type of sugar (Table 5). The assay revealed the total carbohydrate content of the gel and was calibrated with the free sugars. When the same type of glycomonomer is used, the amount of incorporated sugar is not strongly influenced by different synthesis parameters such as the amount of stabilizer, crosslinker and initiator, the type of comonomer and crosslinker and the method of initiation. Hence, the sugar content is very similar, independent of glycogel size and crosslinking density. This means that the incorporation of the glycan is solely controlled by the polymerization kinetics of the monomer and not dramatically affected by the actual reaction parameters. On the other hand, a striking difference in sugar content is observed when different glycomonomers are examined. On average, the fucose glycogel contains the highest sugar amount with a calculated incorporation of up to 70% of the initially used glycomonomer, followed by lactose (up to 40%) and melibiose (up to 25%) glycogel. This coincides with the aforementioned fast reaction of FucMAm as it is more hydrophobic and therefore might react similar to and with NiPAm in contrast to the very hydrophilic lactose and melibiose glycomonomers.

### 2.3. Inhibition Studies with Plant Lectins

The aim of this study is to synthesize glycogels with full functionality in means of glycan binding. Therefore, it is important to prove the accessibility of the saccharide units for lectins. For screening the synthesized glycogels, we chose three plant lectins as representatives for melibiose, lactose and fucose binding lectins, respectively—Jacalin [51], *Erythrina cristagalli* lectin (ECL) [52] and *Ulex europaeus* agglutinin I (UEA I) [53]. In ELISA-type inhibition assays, the nanogels were applied as inhibitors for lectin binding to an immobilized ligand.

**Figure 3:**
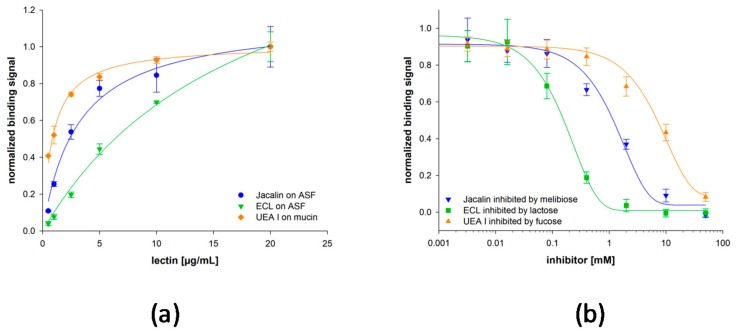
Binding and inhibition of chosen plant lectins to immobilized glycoproteins. (**a**) The lectins Jacalin and *Erythrina cristagalli* lectin (ECL) show binding to immobilized asialofetuin (ASF), whereas *Ulex europaeus* agglutinin I (UEA I) binds to immobilized mucin from porcine stomach. (**b**) The three lectins bind glycan mediated and are inhibitable by appropriate saccharides. Typical inhibition curves are shown for Jacalin inhibited by melibiose, ECL inhibited by lactose and UEA I inhibited by fucose.

First of all, we investigated lectin binding to standard glycoproteins asialofetuin (ASF) and thyroglobulin. ECL and Jacalin bind sufficiently to ASF, whereas for UEA I binding α-fucose residues are necessary. This could be found neither on ASF nor on thyroglobulin (data not shown). As mucins contains fucose units, porcine stomach mucin was tested and found to possess ligands for UEA I. The binding curves of UEA I on mucin as well as of the other two lectins on ASF are shown in Figure 3a. These binding signals are glycan mediated because inhibition with the appropriate sugar was proven (Figure 3b).

**Figure 4:**
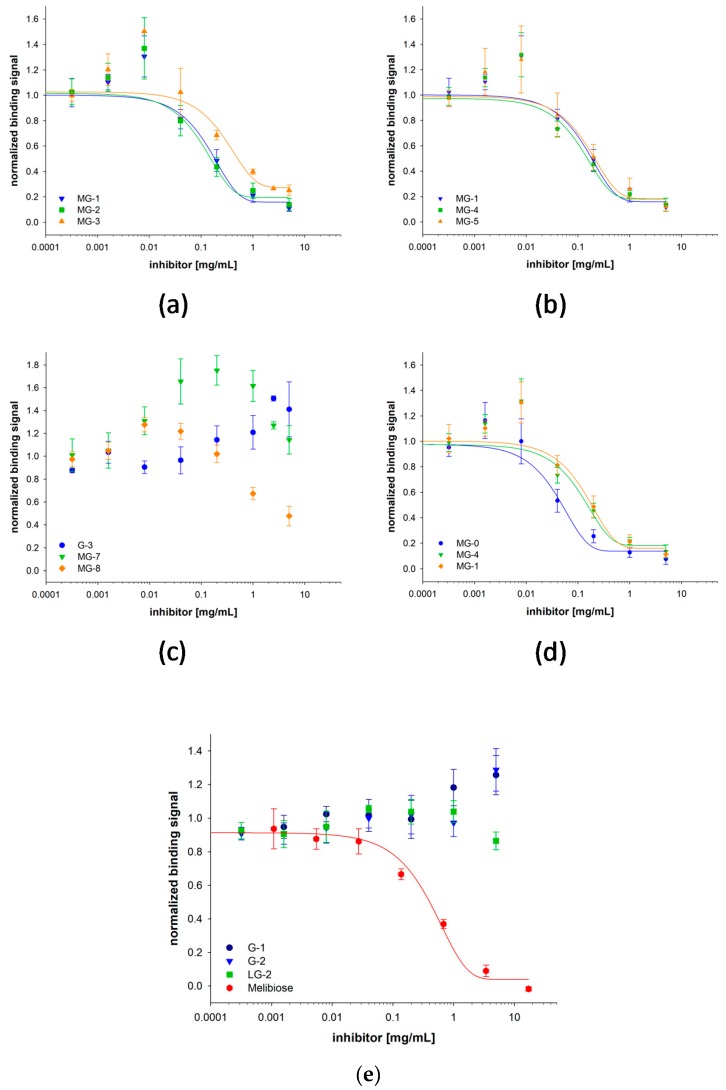
Inhibition of Jacalin binding by glyco-nanogels. Melibiose nanogels were investigated in inhibition studies with Jacalin and compared regarding (**a**) SDS amount during the synthesis, (**b**) initiation method of polymerization, (**c**) type of comonomer and crosslinker, and (**d**) the size of the gels. In (**e**), control nanogels without sugar or containing a non-inhibiting sugar show no inhibition. The complete inhibition by the disaccharide melibiose proved the suitability of the assay.

**Figure 5:**
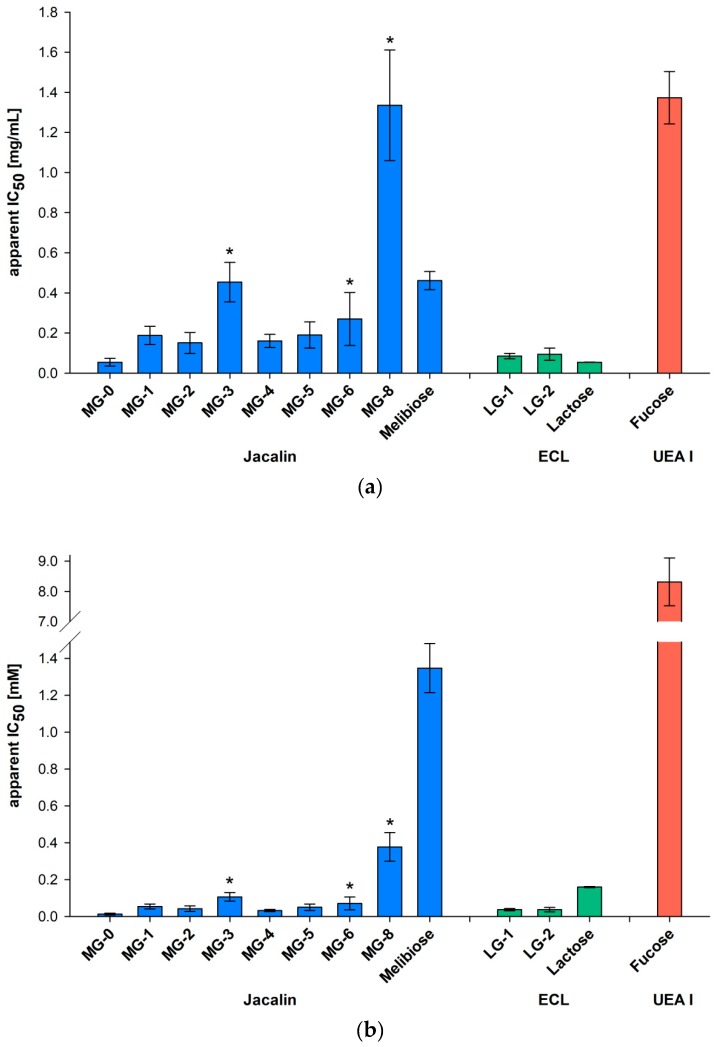
Apparent IC_50_ values of glycogels and free saccharides. The concentration (**a**) (mg/mL) glycogel and (**b**) (mM) sugar content that is needed for half maximal inhibition is shown for melibiose nanogels and free melibiose regarding Jacalin binding, lactose nanogels and free lactose regarding ECL binding and fucose regarding UEA I binding. Gels marked with asterisks did not show complete inhibition.

A set of melibiose containing nanogels was synthesized with different synthesis conditions. The majority of these glycogels inhibited the binding of Jacalin to ASF indicating functional melibiose presentation. The SDS amount during the synthesis had a small influence on the inhibition potential of the glycogels. An increasing amount of SDS seemed to weaken the lectin binding (Figure 4a). However, we tested if this is an effect caused by the denaturing properties of SDS and incomplete removal during dialysis. It turned out that the lectin binding is not affected by SDS at concentrations around 0.1% (data not shown), which represents the highest amount used during synthesis. The initiation method of polymerization is irrelevant for lectin binding, as the inhibition curves for MG-1, MG-4 and MG-5 in Figure 4b are nearly identical. The type of crosslinker as well as the monomer type have the strongest influence. If NiPMAm was used for nanogel synthesis, no lectin inhibition was measured but an elevated binding signal occurred (Figure 4c). This phenomenon was observed for the NiPMAm control G-3 as well as for the melibiose-NiPMAm gel MG-7. Potentially, the lectin interacts with the nanogel somehow but is further able to bind ASF. In general, PNiPMAm as well as PNiPAm are reported to show rather low unspecific protein adsorption [54]. MG-8 has again a NiPAm backbone but EGDMA as crosslinker instead of MBA. EGDMA and MBA exhibit different reaction kinetics which can lead to different sizes and structures in the gel as shown in previous studies with PNiPAm microgels [55]. The change to EGDMA had a negative influence on the functionality and led to weak inhibitory potency as well as incomplete inhibition (Figure 4c and Figure 5). Comparing the glycogels, regarding their size, a positive effect was seen with smaller gels (Figure 4d). MG-4 with approximately 500 nm D_h_ showed slightly better inhibition than MG-1 with approximately 700 nm. But the highest inhibitory potency among all melibiose nanogels had MG-0 that was the smallest by far (approximately 150 nm D_h_). The smaller the particle, the larger the surface and that means, in this case, a higher density of glycans. We found the highest multivalent effect of these smallest nanogels reaching an IC_50_ value of 0.05 mg/mL which is a threefold higher inhibition value than the average of all larger nanogels (see Figure 5a).

Noticeably, in the presence of glycogels of low concentrations (<0.01 mg/mL) the binding of Jacalin to ASF is enhanced, indicated by a higher fluorescence signal than the value for lectin binding without glycogel. Jacalin is a tetrameric lectin and thus, it is able to crosslink the glycogel with the glycoprotein immobilized on the surface [51,56,57]. In this way, the overall amount of glycans on the surface increases and it is possible that more lectins bind to the crosslinked nanogels, resulting in a higher fluorescence being measured. For calculating the IC_50_ values, these data points were neglected.

We also included NiPAm controls without sugar content in our binding study. NiPAm nanogels of small size (G-2) as well as of larger size (G-1) did not inhibit the Jacalin binding (Figure 4e). The same goes for a lactose containing nanogel—no inhibition was observed. Taking these findings together, the synthesized melibiose nanogels contain melibiose units fully functional for lectin binding. The averaged IC_50_ value of melibiose nanogels that inhibit Jacalin binding almost completely is 0.15 mg/mL. In comparison, melibiose as free sugar shows an IC_50_ value of 0.5 mg/mL. In consequence, the glycogels show a multivalent effect that is even higher in consideration of the ratio of sugar monomer during the synthesis of approximately 20% compared to the overall monomer amount. After determining the sugar content of the glycogels and calculating the apparent IC_50_ values from mg/mL glycogel into mM sugar amount (Figure 5b), the multivalent effect of all melibiose nanogels is even more emphasized. Due to the small amount of incorporated melibiose during the synthesis, the IC_50_ values are in average approximately 15-times lower than for melibiose. MG-0 as most potent inhibitor shows even 100-fold higher inhibition than melibiose. Despite not complete inhibition for MG-3, MG-6 and MG-8, those IC_50_ values are still lower than melibiose showing again the multivalent character of sugar presentation of the glycogels. The incomplete inhibition of MG-8 might be due to possible differences in the glycogel structure. The lower inhibition strength of MG-3 and MG-6 can also be explained by different structures as we synthesized MG-3 with high concentrations of stabilizer, which influences the size and morphology of the nanogels. The lower crosslinking density in MG-6 determines the morphology of nanogels as well [50]. With regard to one type of glycogel, and therefore the same sugar content, the inhibitory potency seems to be strongly dependent on the morphology of the glycogels.

**Figure 6:**
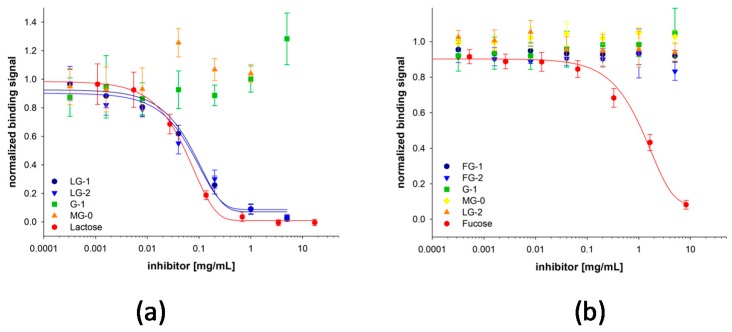
Inhibition of ECL and UEA I binding by glyco-nanogels. (**a**) ECL binding is inhibited by lactose containing nanogels and by free lactose, but not by the control nanogel without sugar and by melibiose nanogel. (**b**) UEA I is only inhibited by free fucose; no synthesized glycogel is bound by UEA I.

The synthesized lactose nanogels were analyzed in binding assays using ECL, a lactose binding lectin [52]. LG-1 and LG-2 are two lactose-containing NiPAm nanogels of very similar syntheses. Both glycogels show complete inhibition of ECL binding to ASF with identical IC_50_ values (Figure 5 and Figure 6a). For ECL, no binding enhancement at low glycogel concentrations is visible. Due to the dimeric structure of ECL, crosslinking of the glycogel with the immobilized glycoprotein is less pronounced and not enough to yield an increased fluorescence signal. G-1, a control PNiPAm nanogel without sugar, as well as MG-0, a melibiose containing nanogel, did not inhibit ECL binding proving the binding specificity to lactose. Moreover, free lactose is a potent inhibitor for ECL with IC_50_ in the same range as the lactose nanogels (Figure 5a). However, due to the limited amount of lactose monomer (approximately 20%) during synthesis, both lactose nanogels contain 0.4 µmol lactose per mg nanogel (Table 5). With IC_50_ values that are over four-times lower compared to free lactose, when regarding the sugar content (Figure 5b), the lactose nanogel also has a multivalent character and led to good inhibition potency. The multivalent effect of the lactose nanogels, however, is less distinct than that of the melibiose nanogels.

The third type of glyco-nanogel we synthesized contains fucose. Therefore, the binding assay was performed with UEA I, a fucose binding lectin [53]. Two different fucose nanogels, FG-1 and FG-2, were tested but no inhibition was observed (Figure 6b). Our synthesis route led predominantly to β-fucose that is rarely found in nature [58]. Thus, it was no surprise that UEA I did not bind to the glycogel. In addition, UEA I is described to be a lectin binding to α-fucose [53]. As seen in Figure 6b, all controls gave the correct results: Nanogel without sugar as well as melibiose and lactose containing nanogels did also not inhibit the UEA I binding, whereas with free fucose selective binding of UEA I to immobilized mucin was proven because it was completely inhibitable. The UEA I binding is the strongest binding to its immobilized glycoprotein among the three tested plant lectins, here. The IC_50_ value of fucose for UEA I binding is three-times (six-times for mM value) higher than melibiose for Jacalin binding and 25-times (50-times for mM value) higher than lactose for ECL binding (Figure 5).

### 2.4. Influence on Pseudomonas aeruginosa

In a small preliminary study, we investigated the influence of the glycogels on the growth of PA. For this, gels were selected by the aforementioned lectin-assay and PA was incubated for 24 h with MG-0, MG-4, LG-1, FG-1 and G-2. FG-1 was chosen despite the fact no binding was detected in the lectin studies, because reports suggest that LecB is capable of binding β-fucose moieties [35,43,48]. The gel concentration was kept at 2 mg mL^−1^ in the cultivation broth and as additional control, we used unmodified melibiose and fucose (2 mg mL^−1^ each).

**Figure 7:**
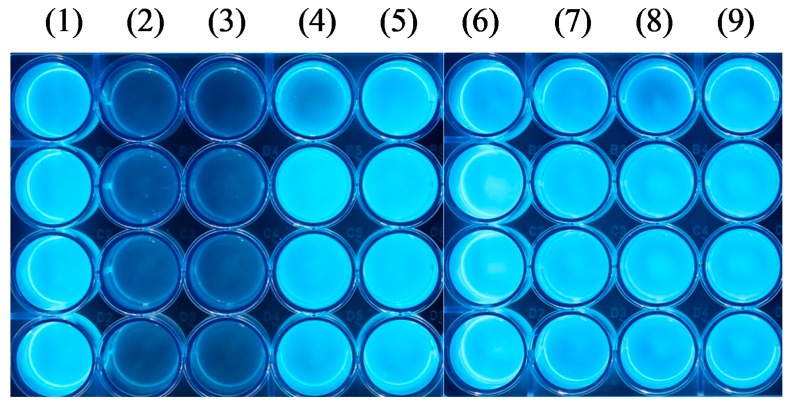
Fluorescence images of *Pseudomonas aeruginosa* (PA) incubated with glycogels. The fluorescence is caused by the secreted fluorescent siderophore pyoverdin. Note the strong decrease in fluorescence signal in the wells containing FG-1. All samples were run as quadruples. Each well in a column is treated identically. (1): MG-0; (2): MG-0 and FG-1; (3): FG-1; (4): LG-1; (5): No gel; (6): MG-4; (7): G-2; (8): Unmodified melibiose and fucose; (9): Same as (5).

In this first study, we focused on the secretion of the fluorescent siderophore pyoverdin, which is an essential virulence factor of PA [59]. Pyoverdin is involved in various processes, including regulation of other virulence factors as well as the enabling the formation of biofilms, which decreases the sensitivity of PA towards antibiotics [60,61]. Figure 7 shows a fluorescence image of the 24-well plates with PA and gels. 

Surprisingly, FG-1 decreased the detectable fluorescence based on pyoverdin secretion clearly. All other samples showed no effect in this regard. To the best of our knowledge there is no hint reported so far that fucose-derivatives are somehow influencing the secretion status of pyoverdin in PA. To investigate if there is really a change in pyoverdin secretion or just an antimicrobial activity of FG-1, the PA were subsequently plated on petri dishes and cultivated for 24 h. All samples showed the formation of colonies, which proves that the effect was not due to antimicrobial activity of FG-1 (see Figure S20, Supporting Information). As these are the first results on this interesting topic, we will proceed with setting up biofilm assays with PA and our glycogels as well as a more in-depth investigation on the influence of fucose containing gels on the secretion of pyoverdin.

## 3. Materials and Methods

### 3.1. Materials

All chemicals were purchased from commercial sources. We recrystallized *N-*isopropylacrylamide (NiPAm; 97%, Sigma Aldrich, Taufkirchen, Germany) and *N*-isopropylmethacrylamide (NiPMAm; 97%, Sigma Aldrich) from n-hexane. Water was double deionized by a Milli-Q purification system (18.2 MΩ cm, Millipore Quantum^®^ TEX, Darmstadt, Germany). The crosslinkers *N*,*N*′-methylenebis(acrylamide) (MBA; 99%, Sigma Aldrich) and ethylene glycol dimethacrylate (EGDMA; 98%, Sigma Aldrich), the initiator 4,4′-azobis(4-cyanovaleric acid) (ABCVA; ≥98%, Sigma Aldrich), dichloromethane (CH_2_Cl_2_; for synthesis) and chloroform (CHCl_3_; extra pure) were redistilled before use. d(+)-Melibiose monohydrate (Mel; ≥99%, Carl Roth, Karlsruhe, Germany), l-(−)-fucose (Fuc; ≥99%, Sigma Aldrich), d-lactose monohydrate (Lac; Carbosynth, Compton, United Kingdom), ammonium carbonate ((NH_4_)_2_CO_3_; ≥30.5% NH_3_, extra pure, Carl Roth), dimethyl sulfoxide (DMSO; VWR, Darmstadt, Germany), methacryloyl chloride (purum, dist., ≥97%, Sigma Aldrich), sodium carbonate (≥99%, anhydrous, Carl Roth), tetrahydrofuran (THF; p. a., Chemsolute, Renningen, Germany), acetonitrile (≥99.8%, for preparative HPLC, Carl Roth), hydroquinone (99.5%, Acros Organics, Darmstadt, Germany), diethylether (Et_2_O; p. a., Chemsolute), methanol (MeOH; extra pure), silica gel (high-purity grade, pore size 60 Å, Sigma Aldrich), sodium dodecyl sulfate (SDS; ≥99.5%, blotting-grade, Carl Roth) were used as received.

### 3.2. Methods

#### 3.2.1. Dynamic Light Scattering (DLS) 

We investigated the hydrodynamic diameter by using dynamic light scattering (DLS) (Malvern Zetasier Nano-ZS, Kassel, Germany). Measurements were performed in disposable polymethylmethacrylate cuvettes at a backscattering angle of 173° five times. We chose temperatures of 20 and 50 °C. For the measurements at 20 °C, we let the samples equilibrate for 5 min, and for the measurements at 50 °C, the samples were allowed to equilibrate for 10 min to ensure complete collapse of the glycogels. We measured the hydrodynamic diameter of PNiPAm nanogel G-1 and melibiose gel MG-4 as a function of the temperature, ranging from 20 to 50 °C.

#### 3.2.2. Scanning Electron Microscopy (SEM)

Diluted samples were dropped onto tilted silicon wafers (CrysTec) to let excess liquid drip off. After letting the wafers dry, the samples were sputtered with platinum (4 nm). Images were taken on a GeminiSEM 300 (Fa. Zeiss, Jena, Germany).

#### 3.2.3. Atomic Force Microscopy (AFM) 

AFM analysis was performed on a Bruker Dimension Icon using NanoScope 9.1 (Karlsruhe, Germany) for measurements and NanoScope Analysis 1.5 for image processing. We measured in ScanAsyst air mode using a ScanAsyst air tip with a spring constant of ~0.4 N/m and a resonant frequency of 70 Hz.3.2.4. NMR and ESI MS NMR spectra were recorded on a Bruker Avance 300 MHz Spektrometer (Ettlingen, Germany). Mass spectra were recorded on FlexarTM SQ 300 MS Detector (PerkinElmer, Rodgau, Germany). 

#### 3.2.4. Thermogravimetric Analysis (TGA)

TGA measurements (TGA 500, TA Instruments, Hüllhorst, Germany) were conducted in nitrogen flow (60 mL/min) with heating rate of 10 K/min up to 200 °C. The water content in the gels was determined to be 5–11 wt.%. For analysis and discussion, we used the wet weight of the gels.

### 3.3. Glycomonomers

The glycomonomers were synthesized in a two-step procedure. For the first step, the respective glycoamines were prepared via Kochetkov amination accelerated by microwave irradiation [44,47]. The second step involves the introduction of a polymerizable moiety following a modified procedure of Ghadban et al. [45].

#### 3.3.1. Synthesis of Glycosylamines

The saccharide was dissolved in solvent and ammonium carbonate was added. The respective amounts of reactants and solvents used are listed in Table 6. Afterwards the reaction mixture was heated in the microwave reactor (START 1500 rotaPREP, MSL, Leutkirch, Germany) to 40 °C for 90 min under stirring. The mixture was allowed to cool down and the ammonium carbonate and the solvent were removed by rotary evaporation at 40 °C under reduced pressure. In case of LacNH_2_, the glycosylamine was precipitated with 40 mL of MeOH after reaction and dried. The crude product was dried in high vacuum and stored at 4 °C. We used the glycosylamines for monomer syntheses without further purification. It has to be noted that residual ammonium carbonate was found in all glycosylamines which could not be removed [46].

#### 3.3.2. Synthesis of Glycosyl Methacrylamides

We dissolved the glycomonomer in a mixture of methanol and Milli-Q water (1:1) and added sodium carbonate. The reaction mixture was cooled in an ice-water bath. Methacryloyl chloride was diluted with THF and dropwise added into the mixture within 10 min under stirring. The reaction was allowed to proceed for further 30–90 min in the ice-water bath (Table 7). Then the volatile solvents were removed by rotary evaporation at 30 °C. The products were purified by silica gel column chromatography (LacMAm: acetonitrile/H_2_O 9:1; MelMAm: acetonitrile/H_2_O 9:1 → 4:1; FucMAm: CHCl_3_/MeOH 5:1), followed by the extraction with diethyl ether in the case of LacMAm. We stabilized MelMAm with hydroquinone (3 ppm) as it tends to polymerize spontaneously. FucMAm was hydrolyzed in 30 mL with 106 mg sodium carbonate (1 mmol; 250 mM) overnight as the NMR spectra showed additional methacrylate peaks. Afterwards, we purified FucMAm by a second column chromatography (CHCl_3_/MeOH 5:1). The products were concentrated and then freeze-dried to obtain white solids (LacMAm: 680 mg, total yield: 57%; MelMAm: 1.12 g, total yield: 18%; FucMAm: 817 mg, total yield: 26%).

LacMAm. ^1^H-NMR (D_2_O, 300 MHz): δ 5.83 (s, 1 H), 5.53–5.67 (m, 1 H), 5.12 (d, *^3^J* = 9.2 Hz, 1 H), 4.51 (d, *^3^J* = 7.7 Hz, 1 H), 3.53–4.01 (m, 12 H), 1.99 (s, 3 H); ^13^C-NMR (DMSO, 75 MHz): δ 184.23 (C), 140.32 (C), 123.87 (CH_2_), 104.46 (CH), 81.09 (CH), 79.40 (CH), 78.02 (CH), 76.94 (CH), 76.70 (CH), 74.09 (CH), 72.91 (CH), 72.52 (CH), 70.13 (CH), 62.62 (CH_2_), 61.44 (CH_2_), 19.26 (CH_3_); ESI MS, *m*/*z* calcd for C_16_H_28_NO_11_: [M + Na]^+^ 432.38, found: 432.11 [M + Na]^+^. 

MelMAm. ^1^H-NMR (300 MHz, D_2_O): δ = 5.81 (s, 1 H), 5.59 (d, *^3^J* = 1.6 Hz, 1 H), 5.08 (d, *^3^J* = 8.9 Hz, 1 H), 5.00 (d, *^3^J* = 3.5 Hz, 1 H), 3.45–4.05 (m, 12 H), 1.98 (s, 3 H); ^13^C-NMR (DMSO, 75 MHz): δ 174.27 (C), 140.28 (C), 123.87 (CH_2_), 99.70 (CH), 81.38 (CH), 78.24 (CH), 77.73 (CH), 73.04 (CH), 72.42 (CH), 70.99 (CH), 69.95 (CH_2_), 62.67 (CH_2_), 19.24 (CH_3_); ESI MS, calcd for C_16_H_27_NO_11_: [M + Na]^+^ 432.38, found: 432.10 [M + Na]^+^.

FucMAm. ^1^H-NMR (300 MHz, D_2_O): δ = 5.78 (1 H, s), 5.56 (1 H, s), 4.98 (1 H, d, *^3^J* = 8.2 Hz), 3.90 (1 H, q, *^3^J* = 6.4 Hz), 3.79–3.94 (1 H, m), 3.61–3.73 (3 H, m), 1.95 (1 H, s), 1.24 (1 H, d, *^3^J* = 6.5 Hz); ^13^C-NMR (DMSO, 75 MHz): *δ* 173.69 (C), 140.22 (C), 123.49 (CH_2_), 81.21 (CH), 74.99 (CH), 73.98 (CH), 72.80 (CH), 70.32 (CH), 19.19 (CH_3_), 17.17 (CH_3_); ESI MS, calcd for C_10_H_17_NO_5_: [M + Na]^+^ 254.24, found: 254.04 [M + Na]^+^.

### 3.4. Synthesis of Nanogels via Precipitation Polymerization 

#### 3.4.1. Synthesis of PNiPAm Nanogel G-1 

The reactants, except the initiator, were dissolved in Milli-Q water in a 100 mL-schlenk flask (Table 8). We purged the solution with nitrogen and equilibrated at 80 °C in an oil bath for 30 min. Afterwards, the reaction was started by adding the initiator in nitrogen countercurrent. We allowed the turbid mixture to cool down after 4 h of reaction. The nanogels were dialyzed against deionized water for several days and then lyophilized to obtain a white solid. 

#### 3.4.2. Synthesis of PNiPAm Nanogel G-2 and PNiPMAm Nanogel G-3 

We dissolved every reactant in Milli-Q water in a 100 mL-schlenk flask or 50 mL-schlenk flask and purged the reaction solution with nitrogen for 30 min (Table 8). The reaction was started by submerging the flask into an 80 °C oil bath. After reaction, we let the slightly turbid mixture cool down and purified the product by dialysis against deionized water for several days, followed by lyophilization. We obtained white solids. 

#### 3.4.3. Synthesis of Melibiose Glycogels MG-1–MG-8

MelMAm (40.9 mg, 0.1 mmol), comonomer (0.4 mmol; 4 eq.), crosslinker, SDS and initiator were dissolved in 5 mL of Milli-Q water (100 mM total monomer concentration) in a 25 mL-schlenk flask. The respective amounts of the chemicals used are listed in Table 9. We purged the solution with nitrogen for 30 min, before submerging the flask into an 80 °C oil bath. For some glycogels, we added an additional amount of initiator after approximately 2 h in nitrogen countercurrent (see t_1_ in Table 9). The reaction was allowed to proceed for a further amount of time (see t_2_ in Table 9). After cooling down the turbid reaction mixture, we purified the glycogel by dialysis against deionized water for several days and lyophilized the product to obtain a white solid. If large aggregated sediments were observed after reaction, the product was filtered through Kimtech Science^®^ precision wipes (Darmstadt, Germany) before freeze-drying.

In case of MG-5, every reactant was dissolved in Milli-Q water with the exception of the initiator. After purging the reaction solution with nitrogen and equilibrating at 80 °C for 30 min, we added the initiator to start the reaction.

#### 3.4.4. Synthesis of Melibiose Glycogel MG-0 

We first tested the precipitation polymerization with a previously synthesized melibiose monomer that was not stabilized with hydroquinone. We dissolved MelMAm (81.8 mg, 0.2 mmol), NiPAm (90.5 mg, 0.8 mmol; 4 eq.), MBA (15.4 mg, 0.1 mmol; 10 mol%), SDS (1.15 mL of a 10 mg/mL stock solution, 4 × 10^−5^ mmol; 4 mM) and ABCVA (0.7 mg, 2.5 × 10^−3^ mmol; 0.25 mol%) in Milli-Q water (9 mL; 100 mM total monomer concentration). We purged the reaction solution with nitrogen for 30 min. Then, we started the reaction by submerging the reaction flask into an 80 °C oil bath. After 2 h, the reaction mixture remained clear. Additional initiator was added and the mixture turned slightly turbid. We let the reaction proceed for further 4 h before letting it cool down. The glycogel was purified by dialysis against deionized water for several days and then freeze-dried. We obtained 99.7 mg of white solid (53%).

#### 3.4.5. Synthesis of Lactose Glycogel LG 

We dissolved LacMAm (40.9 mg, 0.1 mmol), NiPAm (45.3 mg, 0.4 mmol; 4 eq.), MBA (3.9 mg, 2.5 × 10^−2^ mmol; 5 mol%), SDS (288 µL of a 1 mg/mL stock solution, 1.0 × 10^−6^ mmol; 0.2 mM) and ABCVA (2.8 mg, 0.01 mmol; 2 mol%) in Milli-Q water (4.712 mL; 100 mM total monomer concentration) in a 25 mL-schlenk flask. We purged the solution with nitrogen for 30 min. Then the reaction was started by submerging the reaction flask into an 80 °C oil bath. After 20 h of reaction, we let the reaction mixture cool down. We dialyzed the product against deionized water for several days and lyophilized the product to obtain a white solid (72.5 mg, 75%). 

#### 3.4.6. Synthesis of Fucose Glycogels FG-1 and FG-2 

We dissolved FucMAm (23.1 mg, 0.1 mmol), NiPAm (45.3 mg, 0.4 mmol; 4 eq.), MBA (FG-1: 3.9 mg, 2.5 × 10^−2^ mmol; 5 mol%; FG-2: 7.7 mg, 5.0 × 10^−2^ mmol; 10 mol%), SDS (57.67 µL of a 10 mg/mL stock solution, 2.0 × 10^−6^ mmol; 0.4 mM) and initiator in Milli-Q water (5 mL; 100 mM total monomer concentration) in a 25 mL-schlenk flask. We purged the solution with nitrogen for 30 min, before submerging the flask into an 80 °C oil bath. The reaction was allowed to proceed for 4 h. After cooling down, we purified the glycogel by dialysis against deionized water for several days, filtered through Kimtech Science^®^ precision wipes and lyophilized the product to obtain a white solid (FG-1: 41.5 mg, 56%: FG-2: 25.9 mg, 33%). 

### 3.5. Phenol-Sulfuric Acid Assay for Determination of Total Sugar Content

The phenol-sulfuric acid assay was performed similar to a described method [62]. Two different concentrations of glycogels (1.5 and 0.75 mg/mL for lactose and melibiose nanogels, 4.0 and 2.0 mg/mL for fucose nanogels) were prepared in water. 50 µL of sample was thoroughly mixed with 150 µL sulfuric acid (95%, Th. Geyer, Renningen, Germany). Subsequently, 30 µL of 5% phenol (Sigma-Aldrich) was added, followed by mixing. The mixture was incubated at 90 °C for 5 min and let cool down in a water bath for further 5 min. After transferring the solution into a 96-well plate (Carl Roth) the absorption at 490 nm was measured.

For calculating the total sugar amount for each type of saccharide, lactose, melibiose and fucose were used separately for calibration. Control gels without sugar were also measured to prove suitability of the assay.

### 3.6. Lectin Studies

To prove the accessible sugar content of the nanogels, different sugar binding proteins (lectins) were used for binding studies. Fluorescein-labeled lectins were chosen for easy detection: ECL for lactose (β-galactose) binding, Jacalin for melibiose (α-galactose) binding and UEA I for α-fucose binding (all from Vector Laboratories, via BIOZOL Diagnostica Vertrieb GmbH, Eching, Germany).

The lectin binding to the nanogels was proven by an ELISA-type competitive inhibition assay, similar to previously described assays [63,64]. Glycogels that are bound by the lectin inhibit the lectin binding to an immobilized glycoprotein. The standard glycoprotein for ECL and Jacalin is ASF. For UEA, we found good binding to mucin from porcine stomach. The binding of the three lectins to its appropriate ligands was proven in a binding assay varying the lectin concentration.

In microtiter plates (MaxiSorp, Nunc, Wiesbaden, Germany) ASF (100 μL of 5 μg/mL bovine ASF (Sigma-Aldrich)) or mucin (100 µL of 100 µg/mL porcine stomach mucin (Sigma-Aldrich), both in sodium carbonate buffer pH 9.6) was immobilized overnight. After washing with PBS-Tween (0.05% (*v/v*) Tween-20) residual binding sites were blocked with 2% BSA (bovine serum albumin, Carl Roth) in PBS. Wells were washed once with PBS-Tween and twice with lectin buffer (10 mM HEPES, 150 mM NaCl, 0.1 mM CaCl_2_, pH 7.5). Varying concentrations of inhibitor and 5 or 10 µg/mL of lectin were incubated simultaneously for 1 h. Controls without inhibitor and without lectin were performed to indicate minimal and maximal binding, respectively. Wells were again washed with lectin buffer and residual bound lectin was detected by fluorescence read-out at 488/520 nm. Measured data were analyzed using Sigma Plot (Systat software GmbH, 11.0, Erkrath, Germany).

### 3.7. Cultivation of PA

The nanogels (4 mg) were swollen over night at 37 °C under shaking conditions in 1 mL Nutrient Broth (NB, Carl Roth).

Nutrient broth was inoculated with *Pseudomonas aeruginosa* and grown under shaking conditions (110 rpm) at 37 °C overnight. The overnight culture was diluted to an OD600 nm of 0.2 with NB and subsequently diluted with the nanogel suspension in a ratio of 1:2 (nanogel 2 mg/mL, PA OD 0.1). 500 µL of this mixture were plated in a 24-well plate (*n* = 4) (TPP, Techno Plastic Products AG, Trasadingen, Switzerland)) and cultivated under static condition overnight at 37 °C. Then the fluorescence of pyoverdin was detected using UV light (Dark Hood DH-50, BIOSTEP, FELIX 2000, Burkhardtsdorf, Germany).

All samples and the controls (PA in NB) showing fluorescence were diluted to 10^−4^–10^−6^ with PBS (Biochrom AG, Berlin, Germany). Samples without fluorescence were diluted to 10^−2^. Subsequently, 100 µL of each sample was plated on cetrimid agar plates (Carl Roth) and stored overnight (37 °C).

## 4. Conclusions

For the first time, we prepared melibiose, fucose and lactose containing nanogels via precipitation polymerization of NiPAm and glycomonomers. We varied the reactions conditions of the gel production and analyzed the inhibitory potency of the gels in lectin assays. The gels showed sugar dependent inhibition of the lectin binding and a prominent multivalent effect compared to unmodified saccharides. We found that overall the inhibition strength increases with decreasing gel size. Furthermore, the monomer NiPAm and crosslinker MBA are more suitable for these lectin assays than NiPMAm and EGDMA as the latter two themselves influence the binding behaviour. The crosslinker amount influences the yield and the lectin binding differently, depending on the glycomonomer. At the same sugar content, the inhibitory potency seems to be strongly dependent on the morphology of the glycogel. Interestingly, the amount of incorporated sugar is not strongly influenced by the reaction parameters but by the type of glycomonomer. This enables a tuning of the synthesis towards yields, optimized size and morphology without decreasing sugar content in the gels. Fucose containing gels showed no inhibition due to the β-anomeric form of the glycomonomer. However, LecB is reported to bind β-fucose residues. Due to the biocompatibility of the materials a potential use of the gels in alternative treatments of *Pseudomonas aeruginosa* infections could be possible in the future. First trials suggest an influence of fucose gels on the secretion of pyoverdin. Work is in progress to establish biofilm formation assays with PA in the presence of the glycogels as well as a more in-depth investigation of the effect in pyoverdin secretion of β-fucose gels.

## Figures and Tables

**Table 1:** Gels synthesized with varied sodium dodecyl sulfate (SDS) concentrations.

Gel	c(SDS)	Yield	D_h_(50 °C)	PDI
	[mM]	[%]	[nm]	[%]
G-1	0.2	-	218	2.54
MG-1	0.4	67	507	21.2
MG-2	2.0	67	554	26.4
MG-3	4.0	43	1084	56.6

**Table 2:** Hydrodynamic diameters (D_h_) and polydispersity index (PDI) of nanogels.

Gel	D_h_(20 °C)	PDI	D_h_(50 °C)	PDI
	[nm]	[%]	[nm]	[%]
G-1	406	4.30	218	2.54
G-2	101	9.10	54.2	12.9
MG-0	143	30.3	103	23.0
MG-1	669	29.7	507	21.2
MG-2	678	40.7	554	26.4
MG-3	1084	56.6	1084	56.6
MG-4	474	31.5	488	20.4
MG-5	651	22.4	569	18.7
MG-6	436	66.6	328	27.9

**Table 3:** Glycogels synthesized with three different methods of reaction start.

Glycogel	Χ_1_(ABCVA)	Χ_2_(ABCVA)	Yield	D_h_(50 °C)	PDI
	[mol%]	[mol%]	[%]	[nm]	[%]
MG-1	1.0	2.0	67	507	21.2
MG-4	3.0	-	61	488	20.4
MG-5	3.0	-	66	569	18.7

**Table 4:** Comparison of glycogels with different saccharides and crosslinker amounts.

Glycogel	Χ(MBA)	Yield	D_h_(50 °C)	PDI
	[mol%]	[%]	[nm]	[%]
LG-1	5.0	67	507	21.2
MG-6	5.0	46	488	20.4
MG-2	10	66	569	18.7
FG-1	5.0	56	643	51.7
FG-2	10	33	548	65.0

**Table 5:** Amount of incorporated glycomonomer in the glycogels.

Glycogel	Sugar Content	Averaged Sugar Content for Each Glycomonomer Type	Theoretical Sugar Content ^a^	Yield of Incorporated Glycomonomer ^b^
	[µmol/mg]	[µmol/mg]	[µmol/mg]	[%]
LG-1	0.43 ± 0.03	0.41 ± 0.03	1.12	38.3 ± 2.7
LG-2	0.39 ± 0.01		1.16	33.7 ± 0.9
MG-0	0.24 ± 0.02	0.26 ± 0.05	1.16	20.8 ± 1.7
MG-1	0.29 ± 0.06		1.14	25.4 ± 5.3
MG-2	0.28 ± 0.02		1.14	24.5 ± 1.8
MG-3	0.23 ± 0.04		1.14	22.8 ± 3.5
MG-4	0.20 ± 0.05		1.14	18.1 ± 4.5
MG-5	0.26 ± 0.05		1.11	23.5 ± 4.5
MG-6	0.26 ± 0.02		1.12	23.1 ± 1.8
MG-7	0.27 ± 0.02		1.06	25.5 ± 1.9
MG-8	0.28 ± 0.04		1.14	24.5 ± 3.5
FG-1	0.94 ± 0.19	0.98 ± 0.16	1.43	65.6 ± 13
FG-2	1.01 ± 0.13		1.43	70.5 ± 9.1

^a^ Theoretical amount of incorporated glycomonomer if complete turn-over of all relevant components is considered. ^b^ Measured sugar content divided by theoretical sugar content.

**Table 6:** Synthesis details of glycosylamines.

Reactant	n	m	V
	[mmol]	[g]	[mL]
Lactose monohydrate	8.33	3.3	-
DMSO	-	-	12.0
(NH_4_)_2_CO_3_	52.0	5.0	-
Melibiose monohydrate	13.9	5.0	-
H_2_O	-	-	100
(NH_4_)_2_CO_3_	520	50	-
Fucose	9.14	1.5	-
MeOH	-	-	13.0
(NH_4_)_2_CO_3_	78.1	7.5	-

**Table 7:** Synthesis details of glycomonomers.

Reactant	n	m	V
	[mmol]	[g]	[mL]
LacNH_2_	3.17	1.0815	-
Sodium carbonate	12.67	1.3434	-
MeOH/H_2_O (1:1)	-	-	16.4
Methacryloyl chloride	9.5		0.9141
THF	-	-	6.3373
MelNH_2_	13.9	5	-
Sodium carbonate	77.8	8.25	-
MeOH/H_2_O (1:1)	-	-	150
Methacryloyl chloride	42.6		4.77
THF	-	-	35
FucNH_2_	12.2	2	-
Sodium carbonate	68.1	7.22	-
MeOH/H_2_O (1:1)	-	-	132
Methacryloyl chloride	42.5		4.1
THF	-	-	35

**Table 8:** Synthesis details of PNiPAm and PNiPMAm nanogels ^a^.

Nanogel	V(H_2_O)	c(Monomer)	n(Monomer)	Χ(CL)	c(SDS)	Χ(ABCVA)	t_1_	Yield
	[mL]	[mmol/L]	[mmol]	[mol%]	[mmol/L]	[mol%]	[h]	[%]
G-1	50	100	5	5	0.2	0.25	4.0	-
G-2	50	100	5	10	4.0	2.00	22	86
G-3 ^b^	25	100	2.5	5	1.0	2.00	4.5	78

^a^ The molfraction Χ refers to the total monomer amount of substance. Comonomer is NiPMAm if not stated otherwise. Crosslinker is MBA if not stated otherwise. ^b^ Comonomer is NiPMAm. Crosslinker is EGDMA.

**Table 9:** Synthesis details of melibiose glycogels ^a^.

Glycogel	n(Comonomer)	Χ(CL)	c(SDS)	Χ_1_(ABCVA)	t_1_	Χ_2_(ABCVA)	t_2_	Yield ^d^
	[mmol]	[mol%]	[mmol/L]	[mol%]	[h]	[mol%]	[h]	[%]
MG-1	0.4	10	0.4	1	2.0	2	17	67
MG-2	0.4	10	2.0	1	1.7	2	16	67
MG-3	0.4	10	4.0	1	2.0	2	20	43
MG-4	0.4	10	0.4	3	22	-	-	61
MG-5	0.4	10	0.4	3	22	-	-	66
MG-6	0.4	5	1.0	2	24	-	-	46
MG-7 ^b,c^	0.4	5	0.2	2	23	-	-	37
MG-8 ^c^	0.4	10	0.4	1	2	2	17	64

^a^ The molfraction Χ refers to the total monomer amount of substance. Comonomer is NiPMAm if not stated otherwise. Crosslinker is MBA if not stated otherwise. ^b^ Comonomer is NiPMAm. ^c^ Crosslinker is EGDMA. ^d^ Yields were determined once.

## Supplementary Materials

The following are available online, Figure S1: ESI MS spectrum of LacMAm, Figure S2: ESI MS spectrum of MelMAm, Figure S3: ESI MS spectrum of FucMAm, Figure S4: ^1^H NMR spectrum of LacMAm, Figure S5: ^13^C-NMR spectrum of LacMAm, Figure S6: ^1^H-NMR spectrum of MelMAm, Figure S7: ^13^C-NMR spectrum of MelMAm, Figure S8: ^1^H NMR spectrum of FucMAm, Figure S9: ^13^C-NMR spectrum of FucMAm, Figure S10: SEM image of MG-4, Figure S11: SEM image of MG-5, Figure S12: AFM image of G-1, Figure S13: AFM image of MG-0, Figure S14: AFM image of MG-1, Figure S15: AFM image of MG-2, Figure S16: AFM image of MG-4, Figure S17: AFM image of MG-5, Figure S18: AFM image of FG-1, Figure S19: AFM image of FG-2, Figure S20: Cetrimid Agar plates of PA incubated with MG-1 (a and b) FG-1 (c and d).
