# Supplementary material for: Functional Glyco-Nanogels for Multivalent Interaction with Lectins
Source: Molecules. 2019 May 15;24(10):1865. doi: 10.3390/molecules24101865 (PMC6572176; doi:10.3390/molecules24101865)
Supplement: Supplementary file 1 [file molecules-24-01865-s001.pdf]

Supporting information

# Functional Glyco-Nanogels for Multivalent Interaction with Lectins

**Jo Sing Julia Tang [1], Sophia Rosencrantz [1], Lucas Tepper [1], Sany Chea [1], Stefanie Klöpzig [2], Anne Krüger-Genge [2], Joachim Storsberg [2] and Ruben R. Rosencrantz [1,*]**

1 Fraunhofer Institute for Applied Polymer Research IAP, Biofunctionalized Materials and (Glyco)Biotechnology, Geiselbergstr. 69, 14476 Potsdam, Germany
2 Fraunhofer Institute for Applied Polymer Research IAP, Biomaterials and Healthcare, Geiselbergstr. 69, 14476 Potsdam, Germany

Emails: josing.tang@iap.fraunhofer.de, sophia.rosencrantz@iap.fraunhofer.de, lucas.tepper@iap.fraunhofer.de, sany.chea@iap.fraunhofer.de, stefanie.kloepzig@iap.fraunhofer.de, anne.krueger-genge@iap.fraunhofer.de, joachim.storsberg@iap.fraunhofer.de

* Correspondence: ruben.rosencrantz@iap.fraunhofer.de; Tel.: +49-331-568-3203

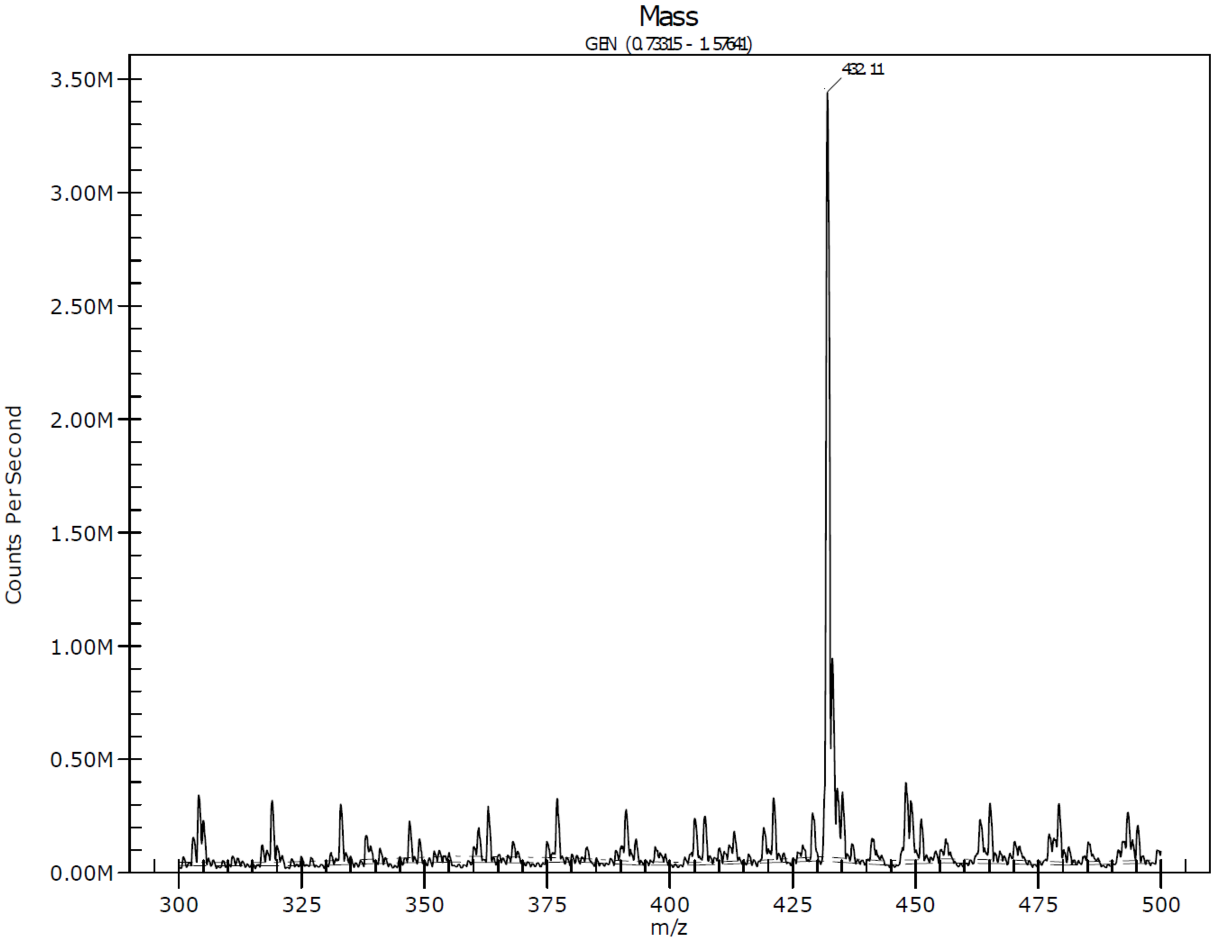


**Figure S1.** ESI MS spectrum of LacMAm.

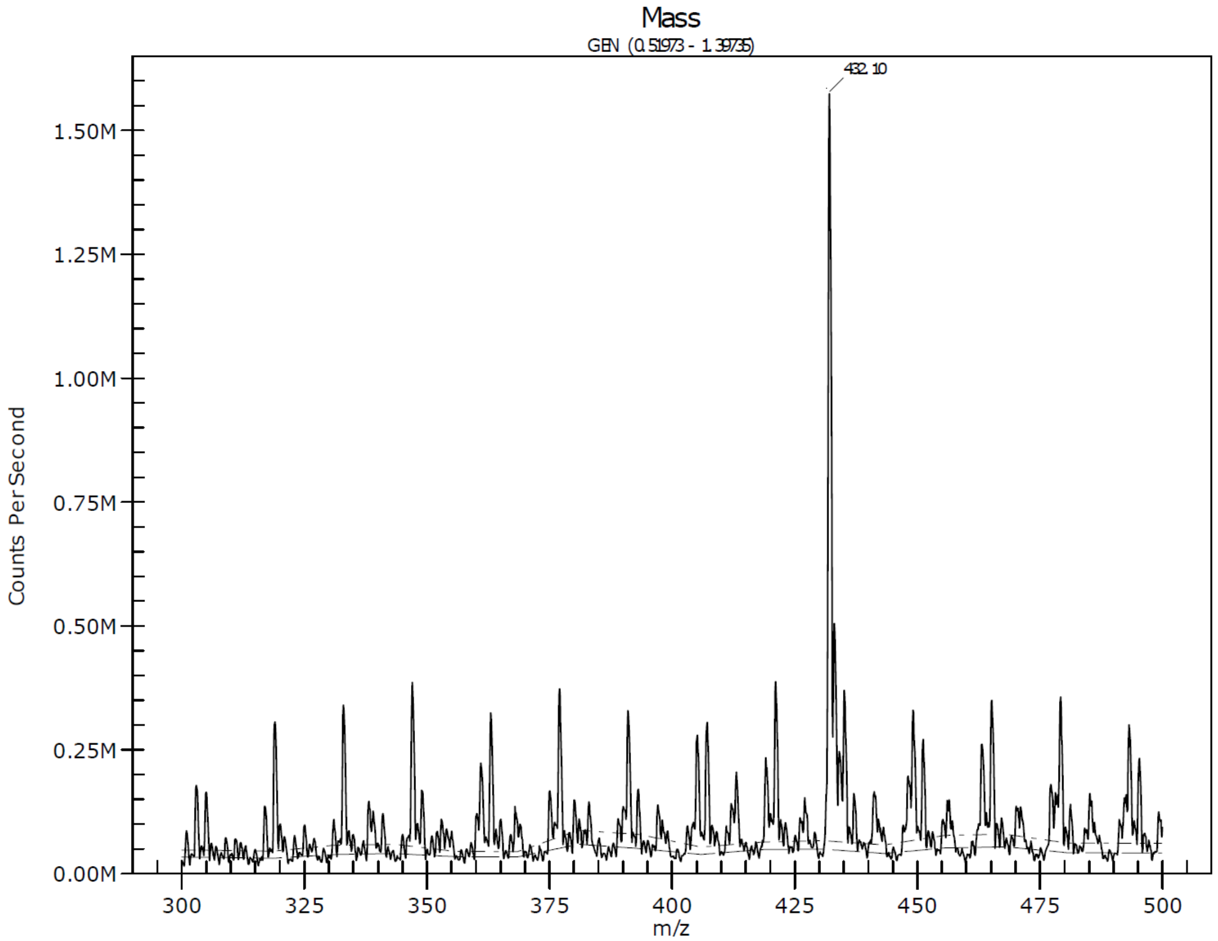


**Figure S2.** ESI MS spectrum of MelMAm.

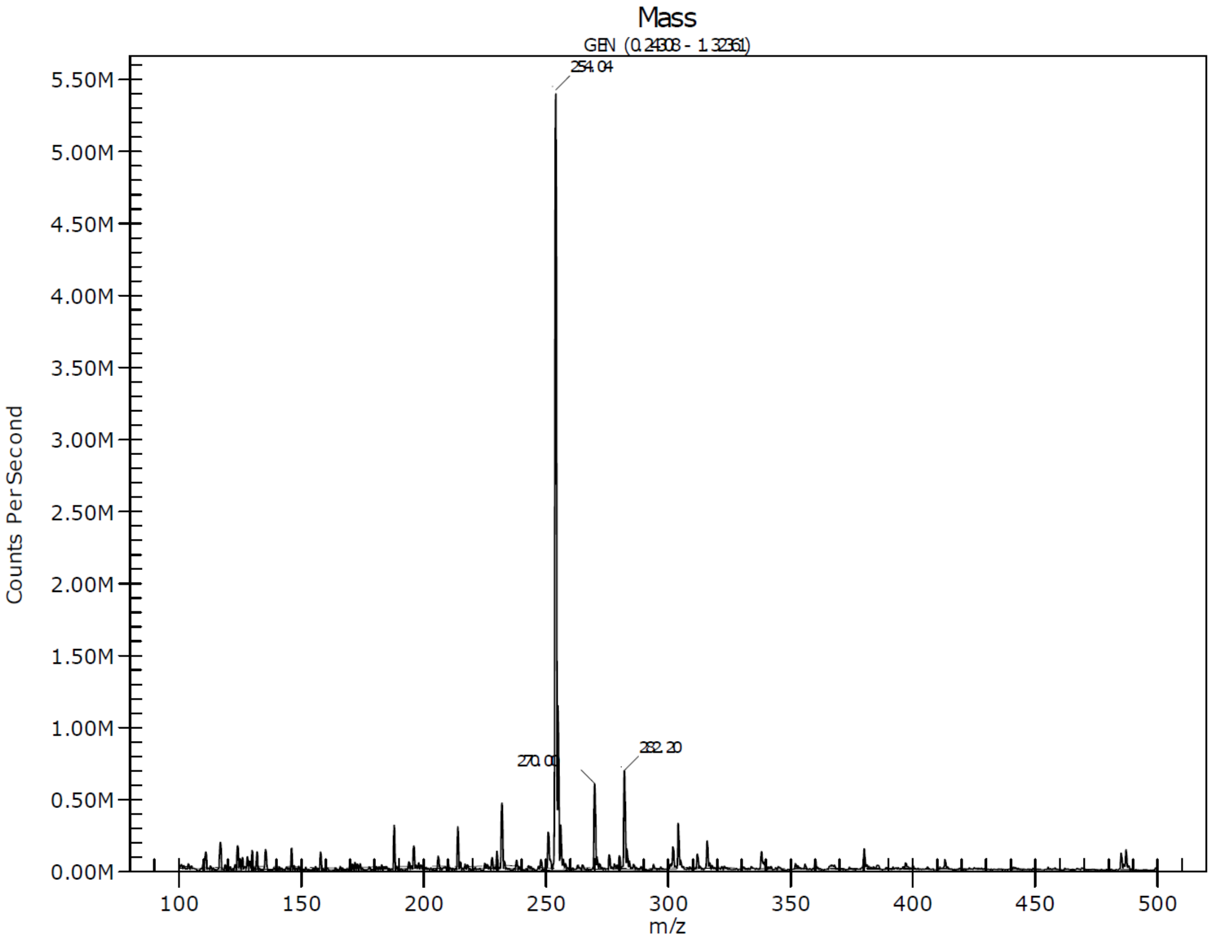


**Figure S3**. ESI MS spectrum of FucMAm.

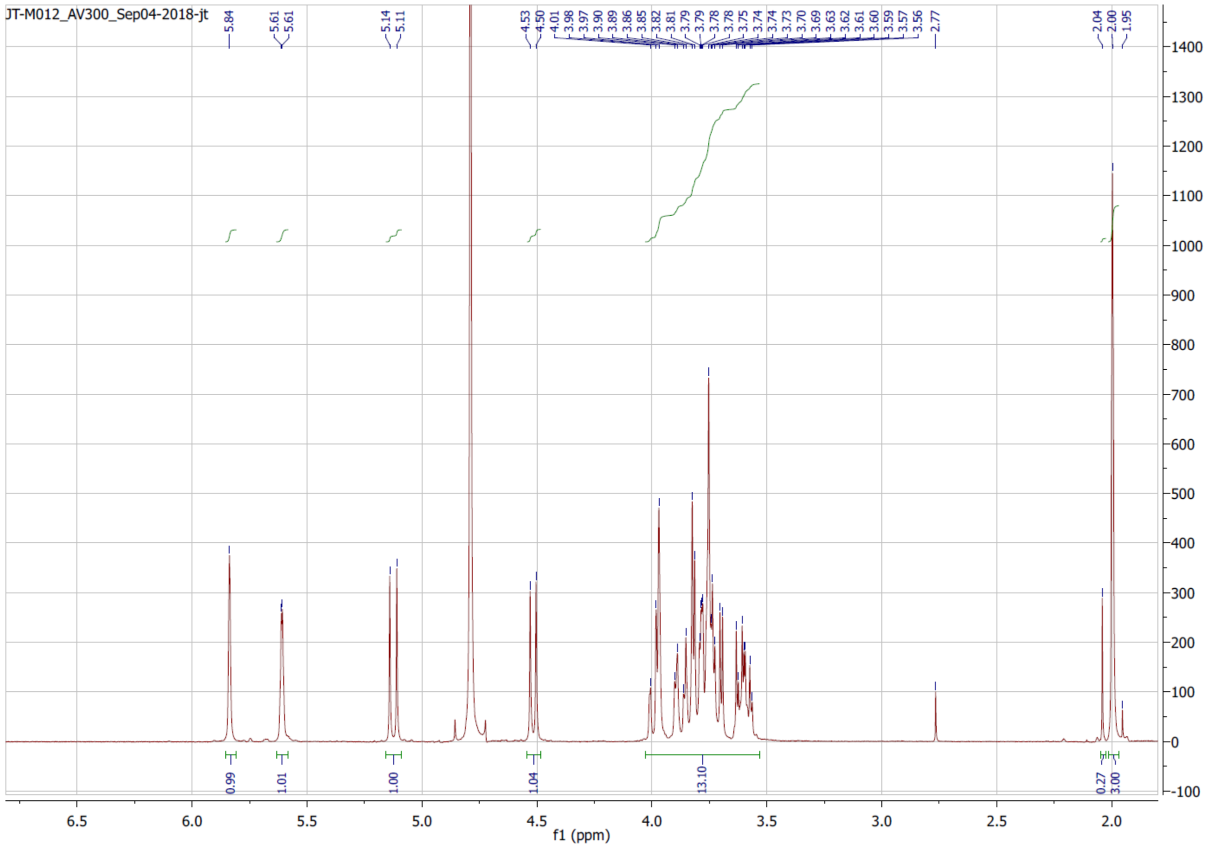

**Figure S4.** $^{1}H$ NMR spectrum of LacMAm.

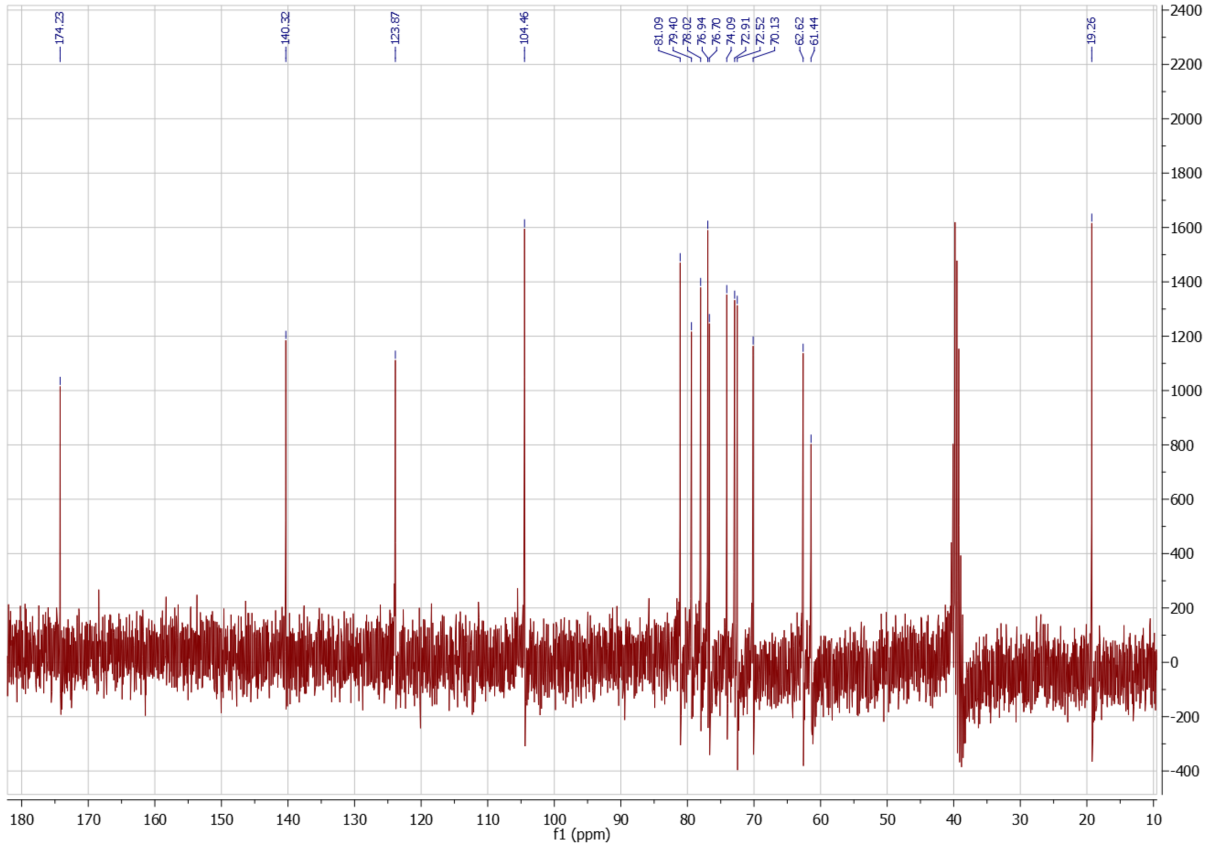

**Figure S5.** $^{13}C$ NMR spectrum of LacMAm.

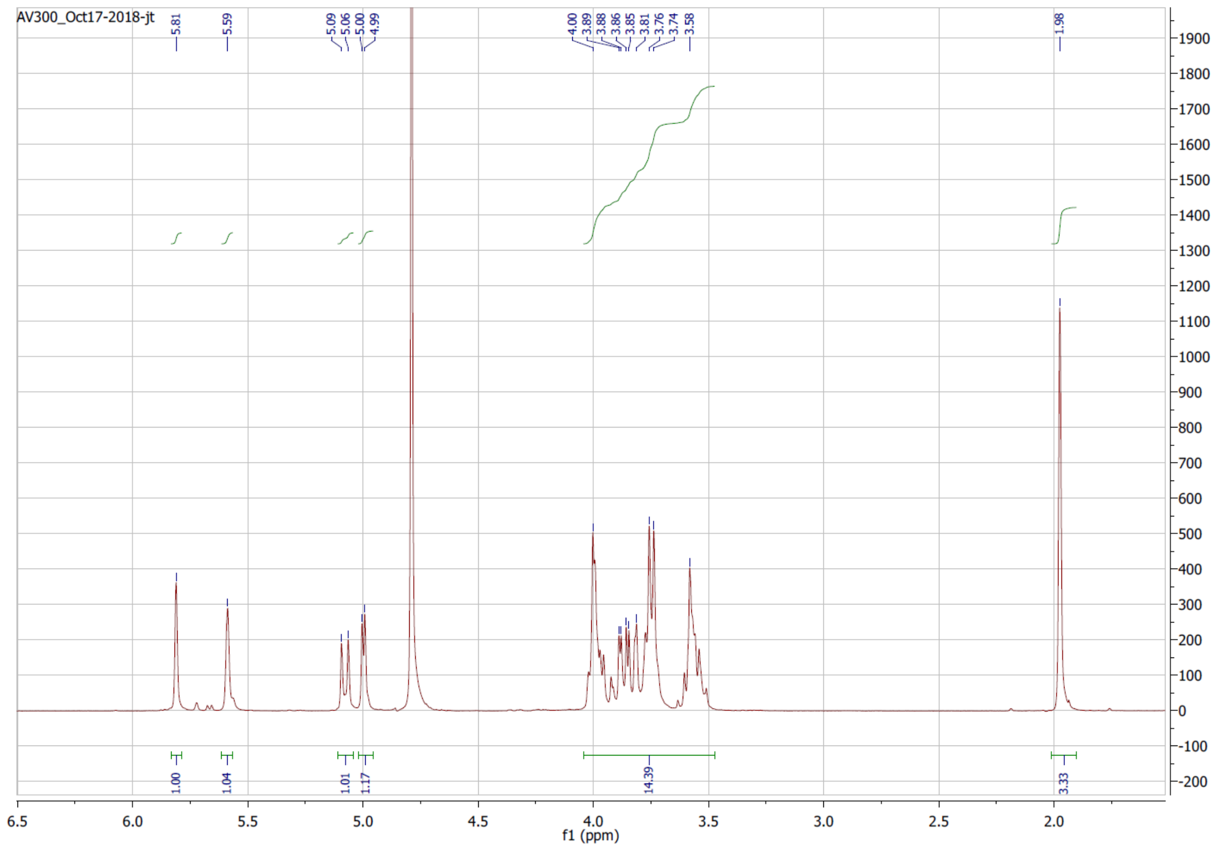


**Figure S6**. ¹H NMR spectrum of MelMAm.

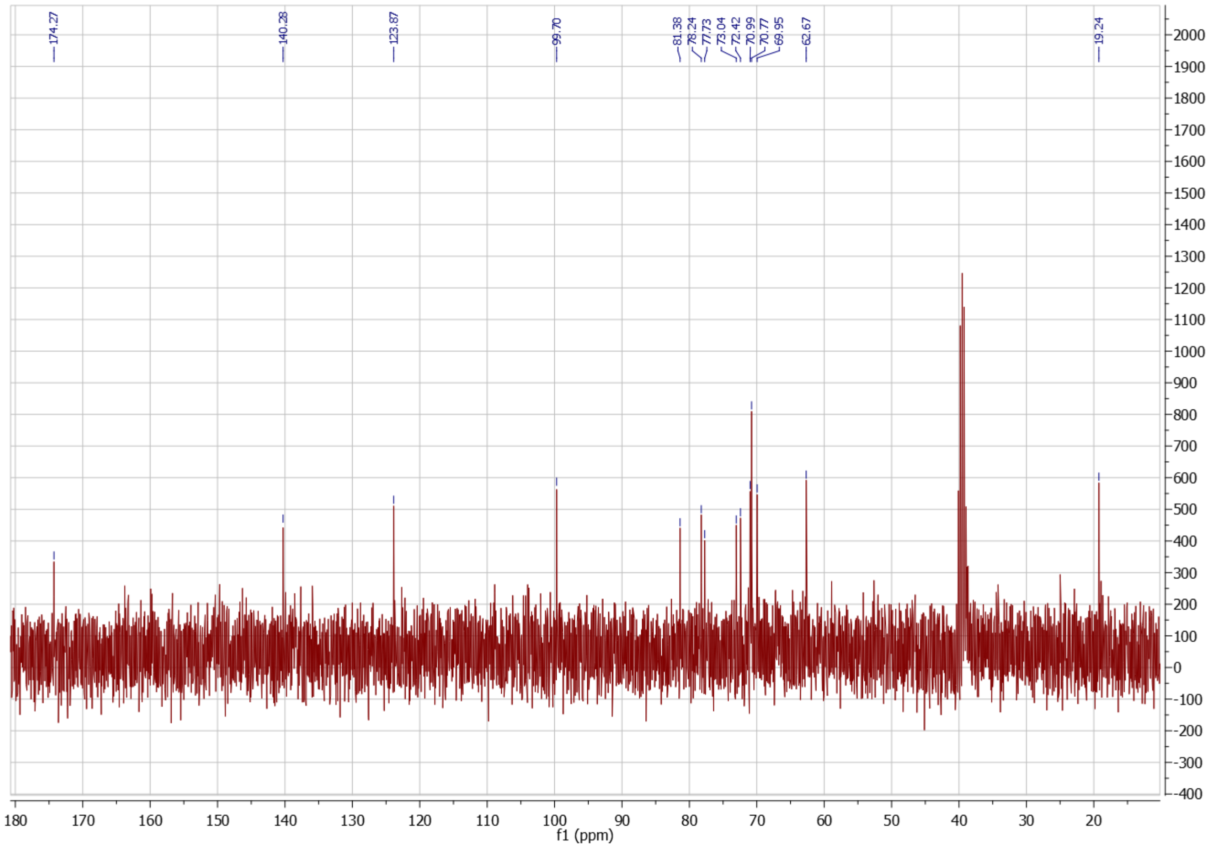


**Figure S7.** ¹³C NMR spectrum of MelMAm.

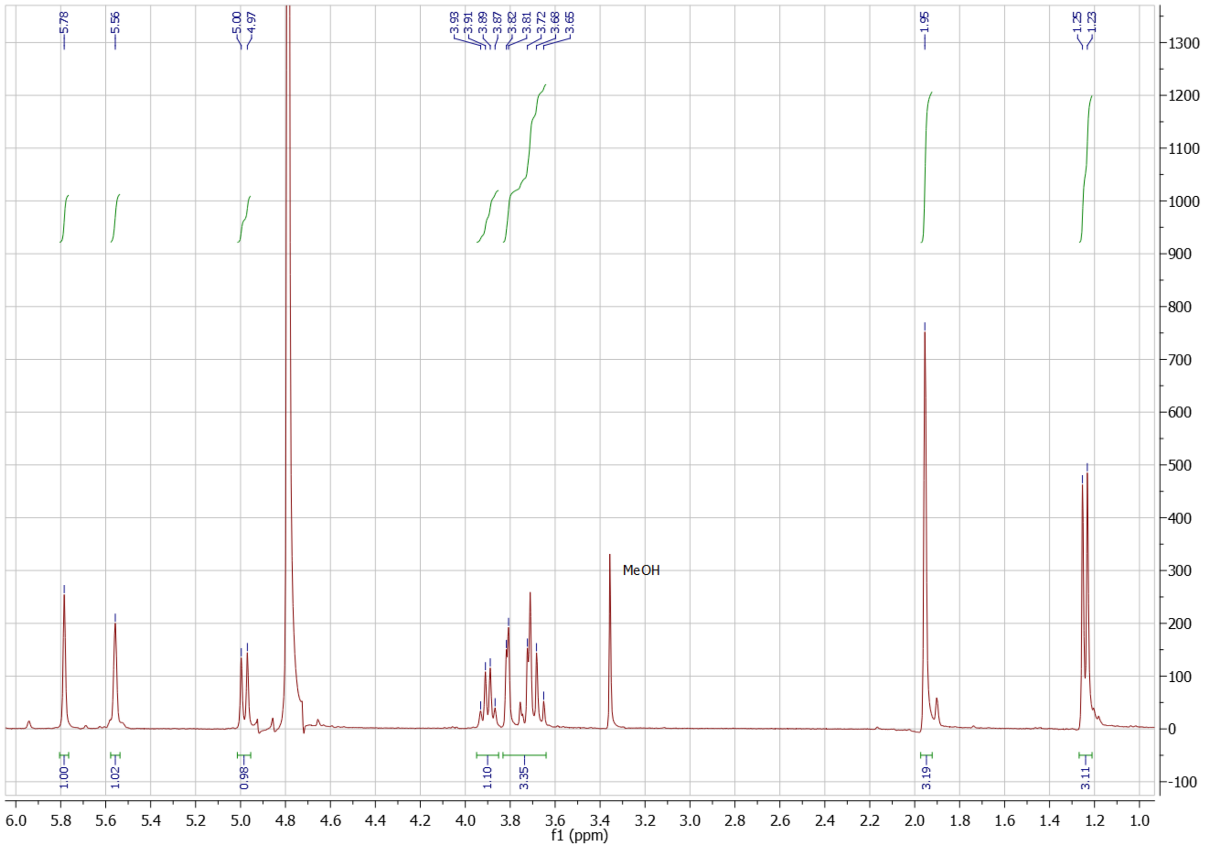


**Figure S8.** $^{1}$H NMR spectrum of FucMAm.

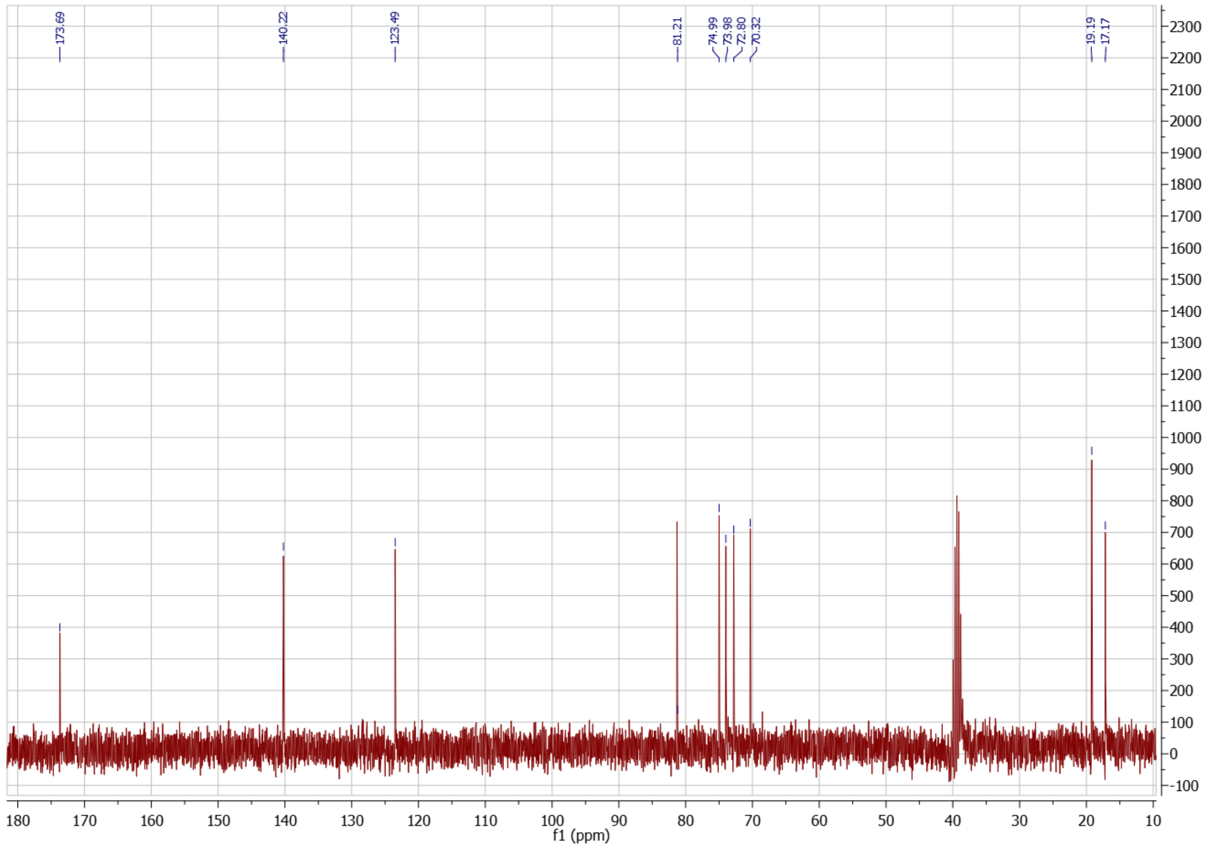


**Figure S9.** $^{13}$C NMR spectrum of FucMAm.

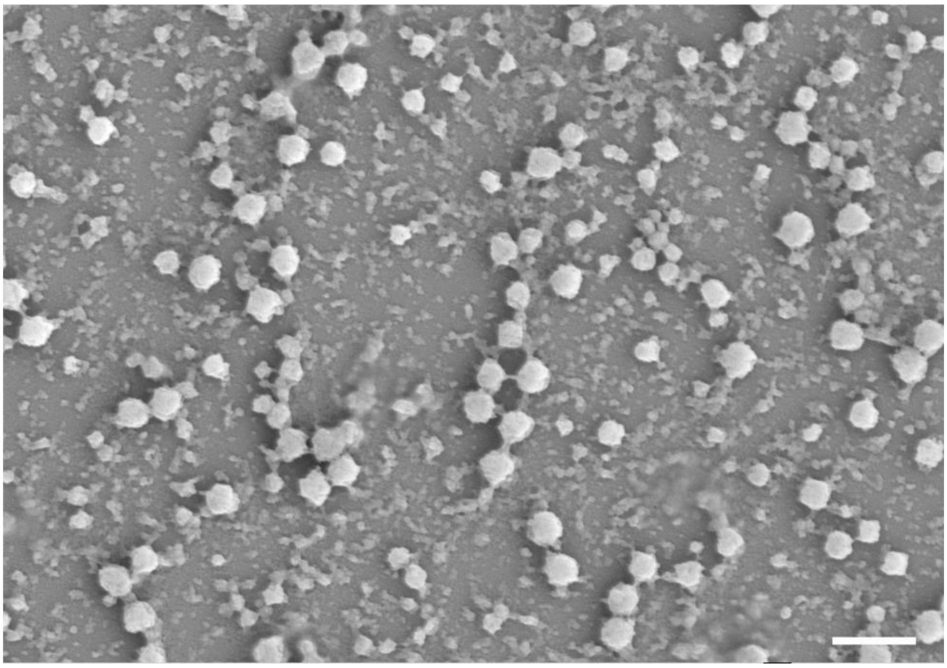

**Figure S10.** SEM image of MG-4. Scale bar: 1 μm.

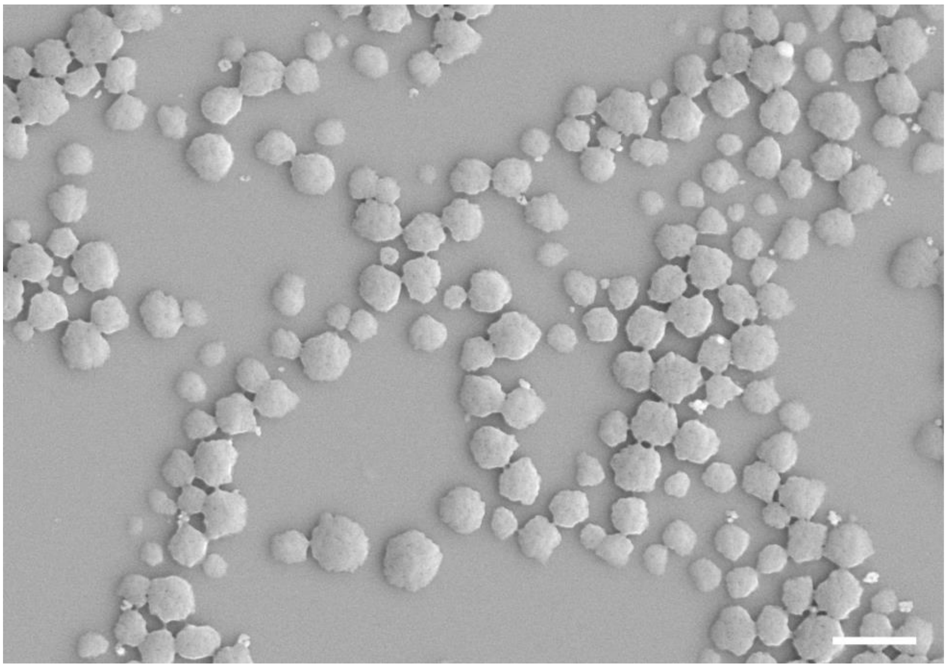

**Figure S11.** SEM image of MG-5. Scale bar: 1 μm.

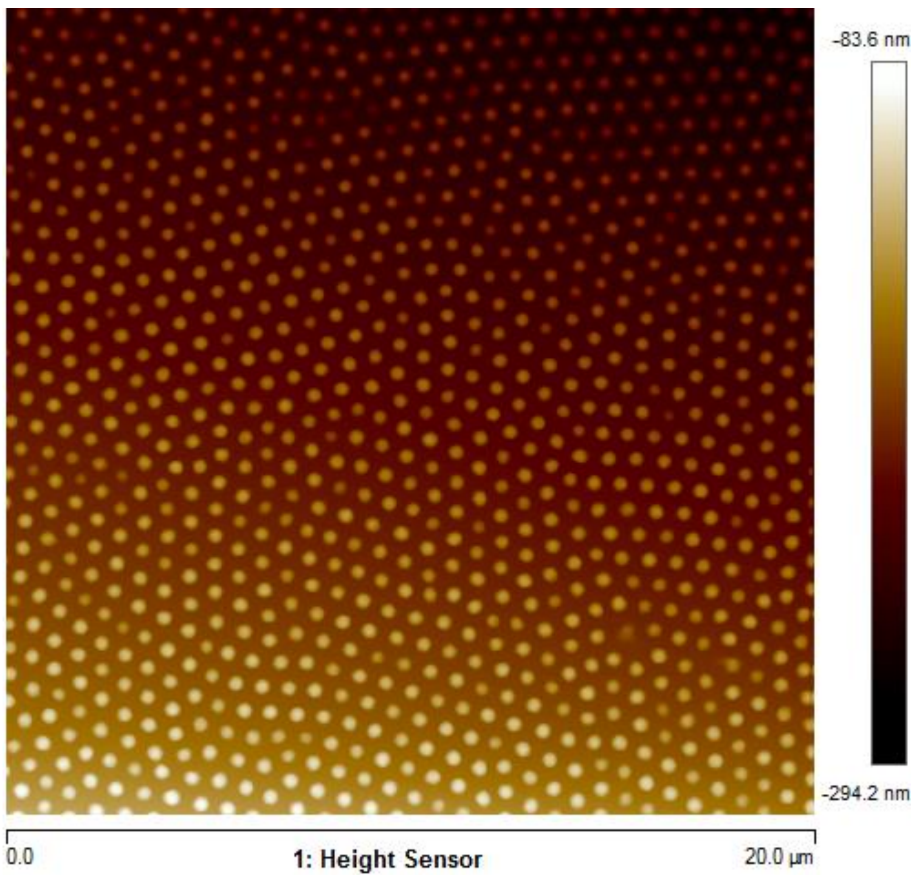


**Figure S12.** AFM image of G-1.

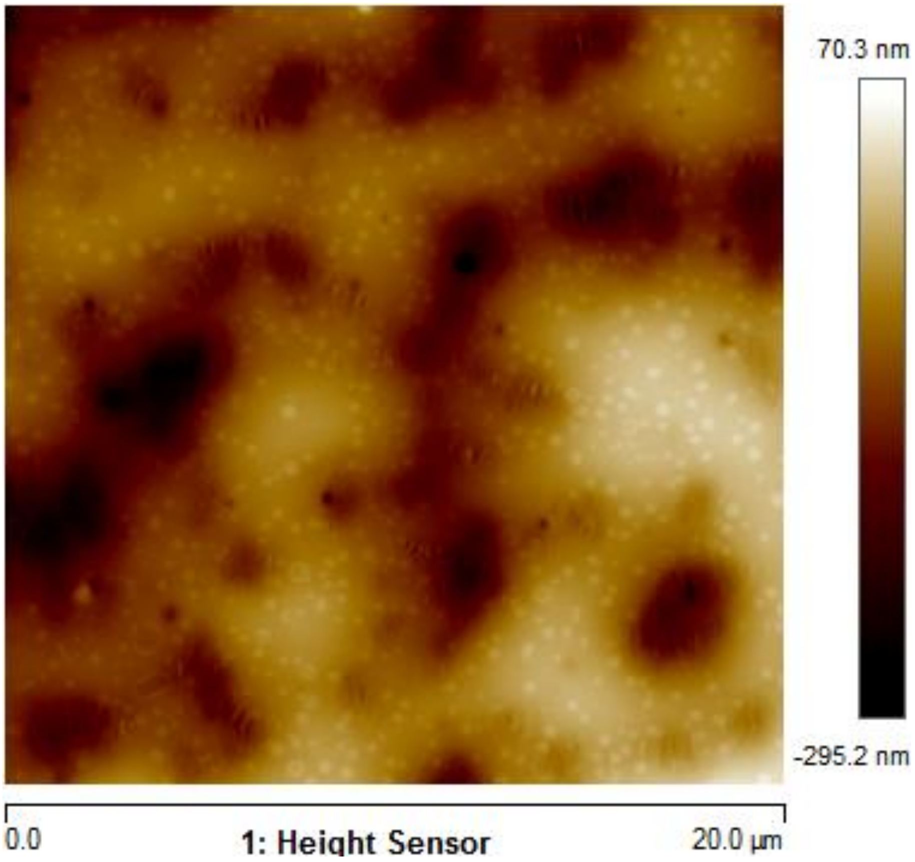


**Figure S13.** AFM image of MG-0.

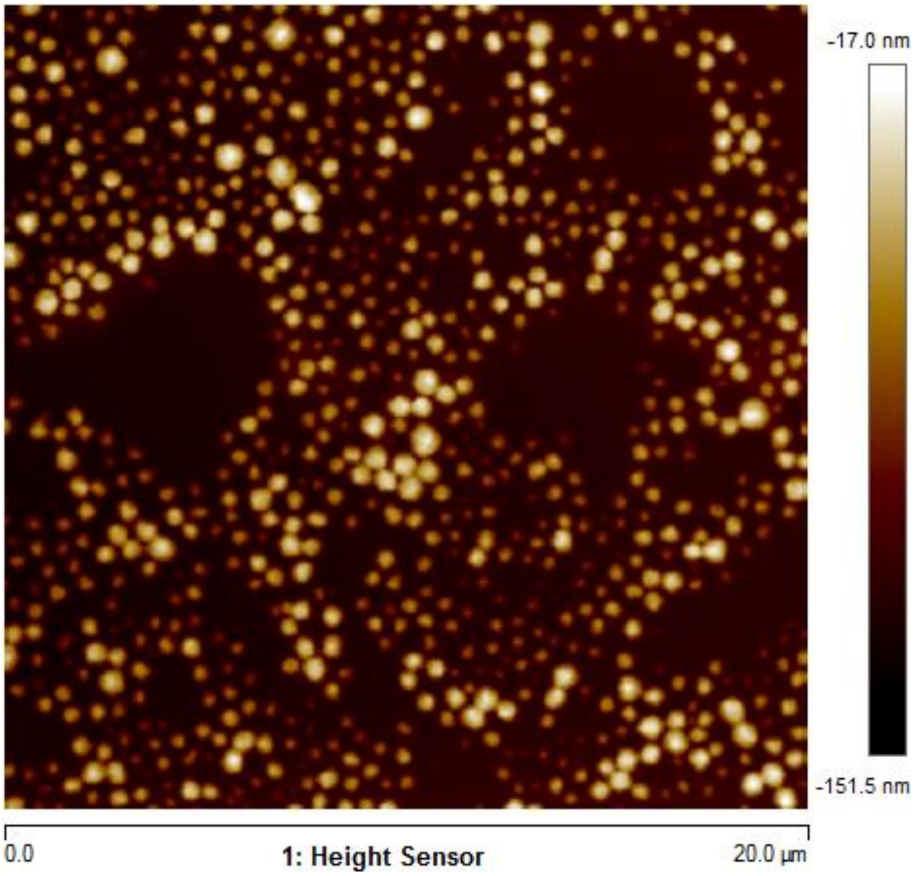


**Figure S14.** AFM image of MG-1.

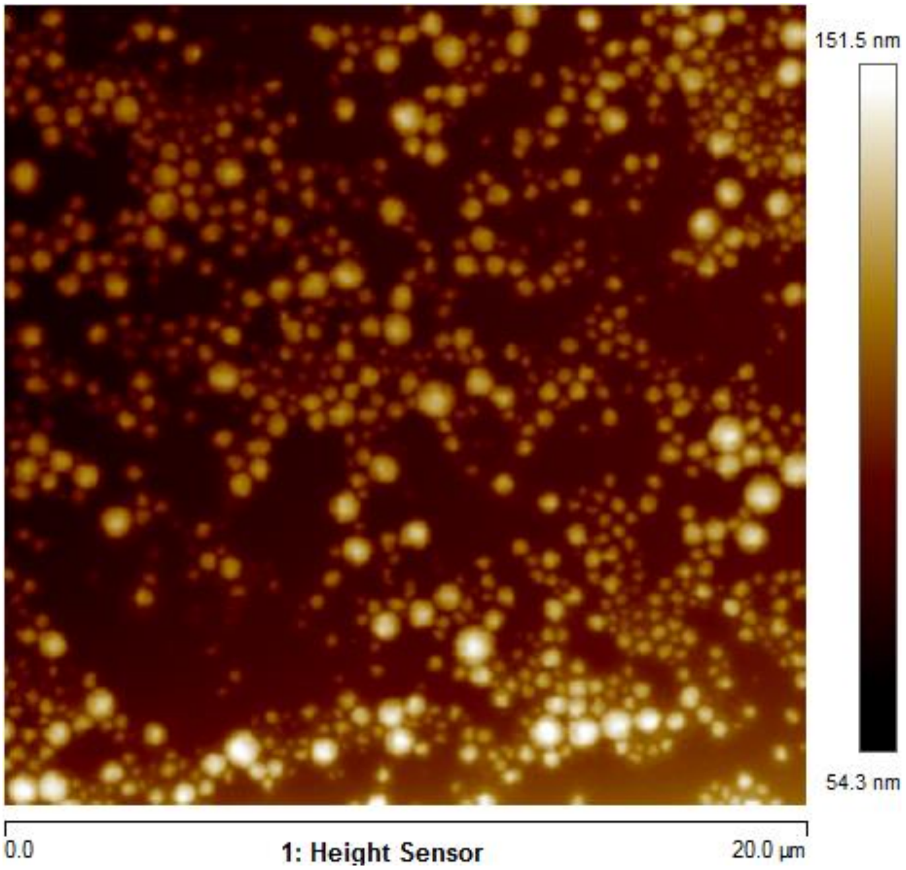


**Figure S15.** AFM image of MG-2.

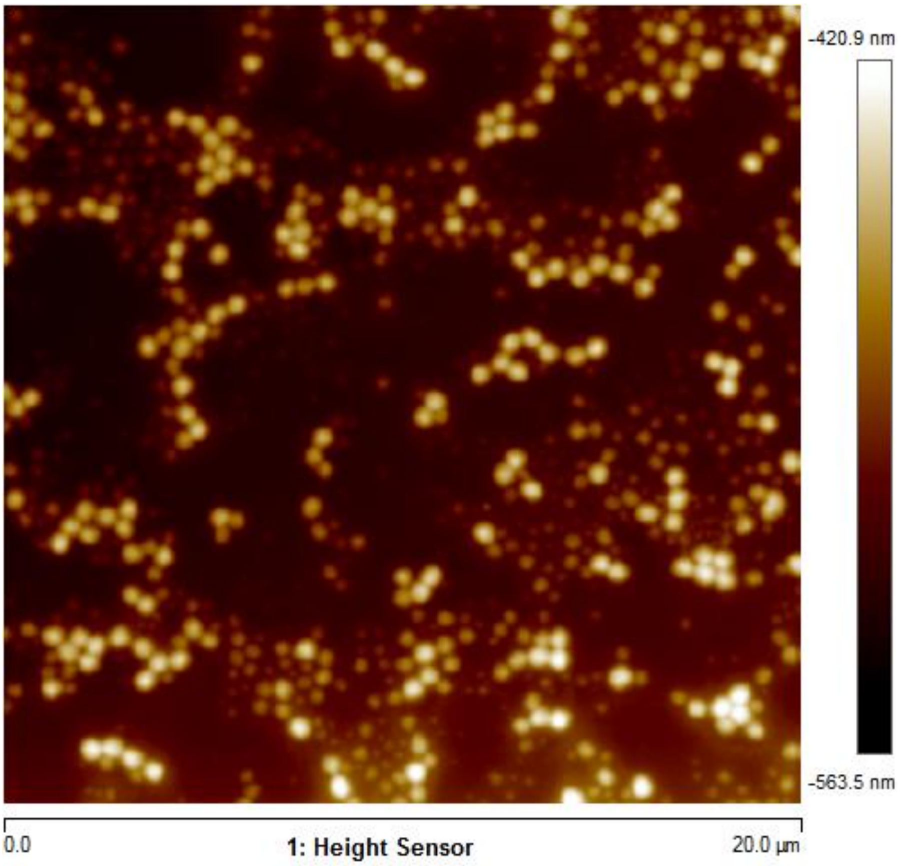


**Figure S16.** AFM image of MG-4.

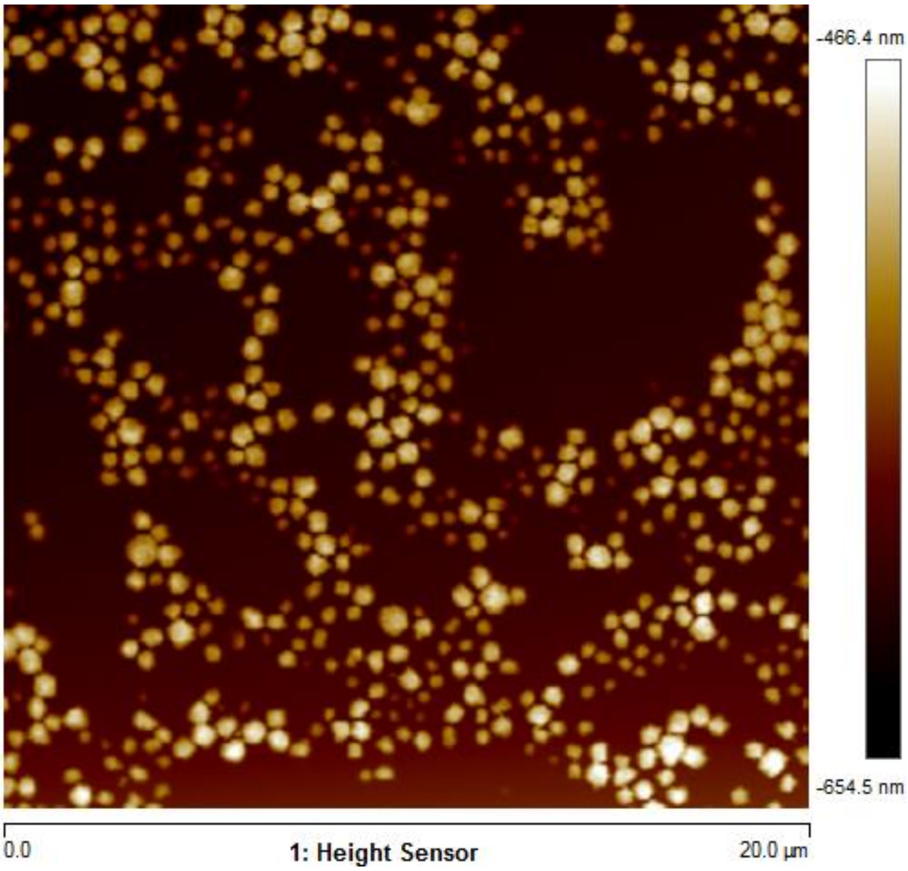


**Figure S17.** AFM image of MG-5.

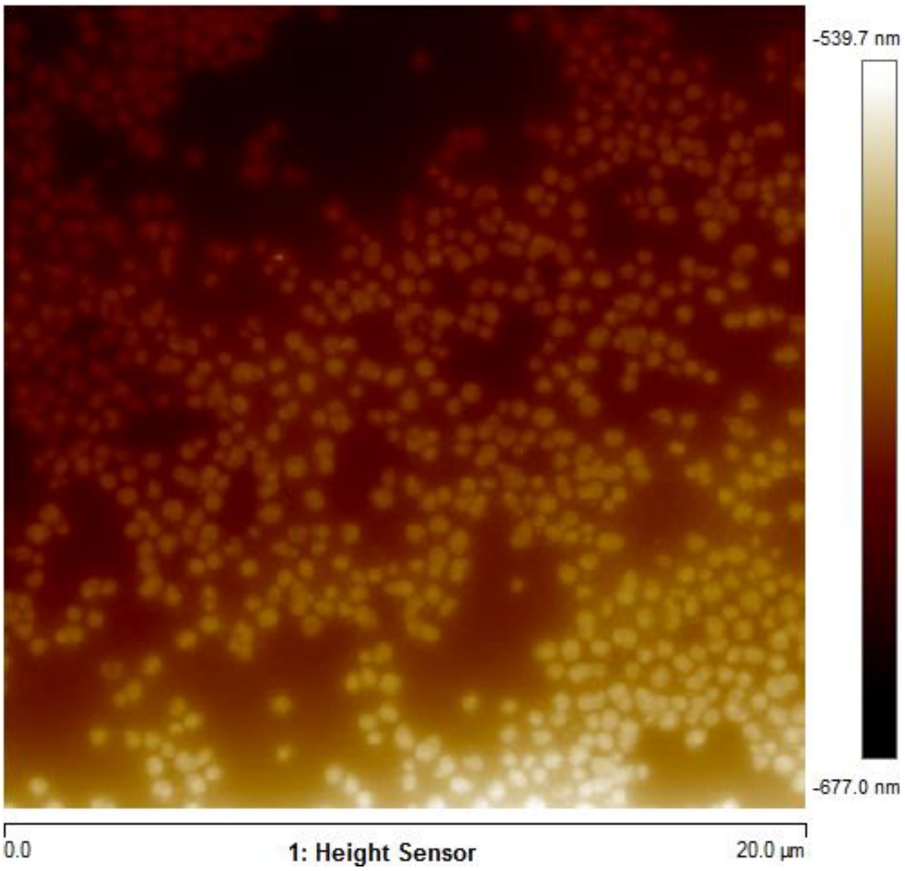


**Figure S18.** AFM image of FG-1.

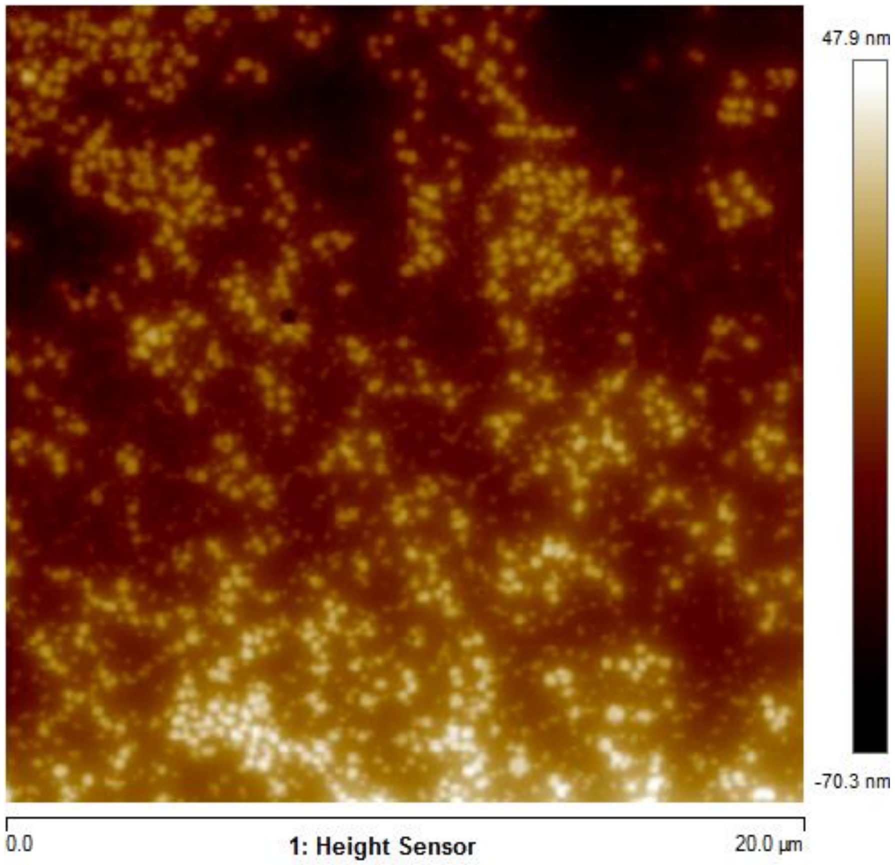


**Figure S19.** AFM image of FG-2.

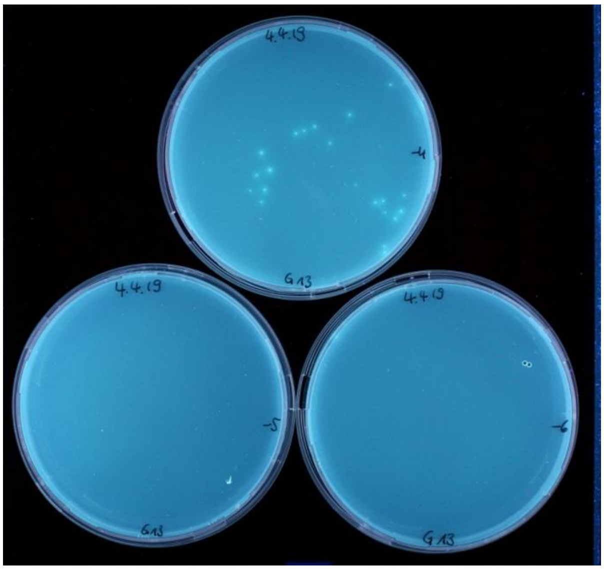


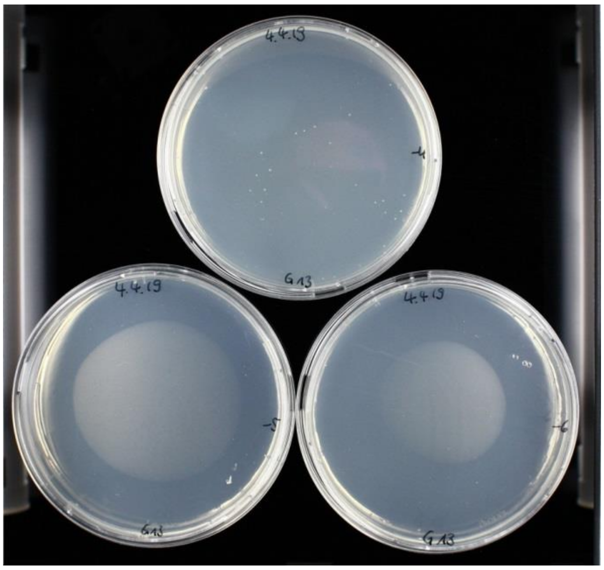


(**a**) (**b**)

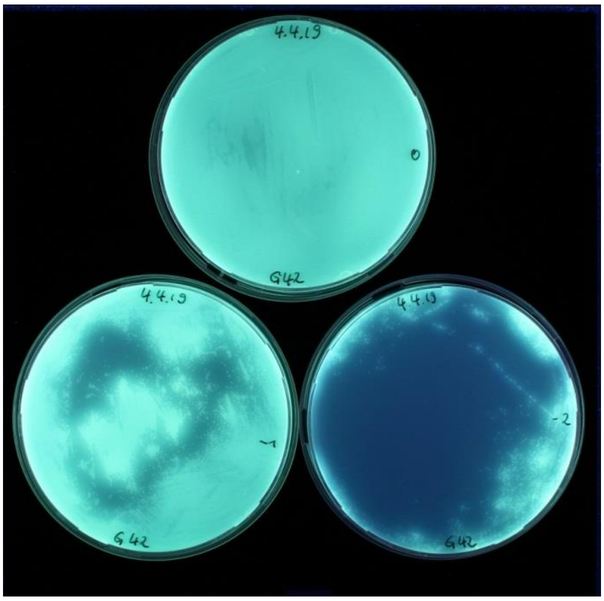


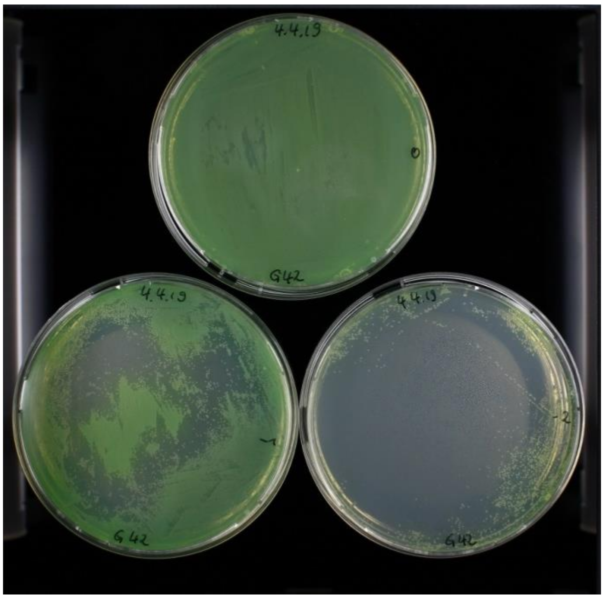


(**c**) (**d**)

**Figure S20.** Cetrimid Agar plates of PA incubated with MG-1 (**a** and **b**) FG-1 (**c** and **d**). (**a**) and (**c**) fluorescence image, (**b**) and (**d**) white light image. FG-1 inhibits pyoverdin, but is not acting antimicrobial. Less colonies are found with MG-1 due to higher dilution. $10^{-4}$ to $10^{-6}$ for MG-1 and undiluted to $10^{-2}$ for FG-1.
